# A Stochastic Multiscale Model That Explains the Segregation of Axonal Microtubules and Neurofilaments in Neurological Diseases

**DOI:** 10.1371/journal.pcbi.1004406

**Published:** 2015-08-18

**Authors:** Chuan Xue, Blerta Shtylla, Anthony Brown

**Affiliations:** 1 Department of Mathematics, Ohio State University, Columbus, Ohio, United States of America; 2 Department of Mathematics, Pomona College, Claremont, California, United States of America; 3 Department of Neuroscience, Ohio State University, Columbus, Ohio, United States of America; University of California, Berkeley, UNITED STATES

## Abstract

The organization of the axonal cytoskeleton is a key determinant of the normal function of an axon, which is a long thin projection of a neuron. Under normal conditions two axonal cytoskeletal polymers, microtubules and neurofilaments, align longitudinally in axons and are interspersed in axonal cross-sections. However, in many neurotoxic and neurodegenerative disorders, microtubules and neurofilaments segregate apart from each other, with microtubules and membranous organelles clustered centrally and neurofilaments displaced to the periphery. This striking segregation precedes the abnormal and excessive neurofilament accumulation in these diseases, which in turn leads to focal axonal swellings. While neurofilament accumulation suggests an impairment of neurofilament transport along axons, the underlying mechanism of their segregation from microtubules remains poorly understood for over 30 years. To address this question, we developed a stochastic multiscale model for the cross-sectional distribution of microtubules and neurofilaments in axons. The model describes microtubules, neurofilaments and organelles as interacting particles in a 2D cross-section, and is built upon molecular processes that occur on a time scale of seconds or shorter. It incorporates the longitudinal transport of neurofilaments and organelles through this domain by allowing stochastic arrival and departure of these cargoes, and integrates the dynamic interactions of these cargoes with microtubules mediated by molecular motors. Simulations of the model demonstrate that organelles can pull nearby microtubules together, and in the absence of neurofilament transport, this mechanism gradually segregates microtubules from neurofilaments on a time scale of hours, similar to that observed in toxic neuropathies. This suggests that the microtubule-neurofilament segregation can be a consequence of the selective impairment of neurofilament transport. The model generates the experimentally testable prediction that the rate and extent of segregation will be dependent on the sizes of the moving organelles as well as the density of their traffic.

## Author Summary

The shape and function of axons is dependent on a dynamic system of microscopic intracellular protein polymers (microtubules, neurofilaments and microfilaments) that comprise the axonal cytoskeleton. Neurofilaments are cargoes of intracellular transport that move along microtubule tracks, and they accumulate abnormally in axons in many neurotoxic and neurodegenerative disorders. Intriguingly, it has been reported that neurofilaments and microtubules, which are normally interspersed in axonal cross-sections, often segregate apart from each other in these disorders, which is something that is never observed in healthy axons. Here we describe a stochastic multiscale computational model that explains the mechanism of this striking segregation and offers insights into the mechanism of neurofilament accumulation in disease.

## Introduction

Axons are long slender projections of nerve cells that permit fast and specific electrical communication with other cells over long distances. The ability of nerve cells to extend and maintain these processes is critically dependent on the cytoskeleton, which is a dynamic scaffold of microscopic protein polymers found in the cytoplasm of all eukaryotic cells. The axonal cytoskeleton comprises microtubules, intermediate filaments called neurofilaments, and microfilaments. Microtubules and neurofilaments are both long polymers that align in parallel along the long axis of the axon, forming a continuous overlapping array that extends from the cell body to the axon tip [1, 2]. Microtubules are stiff hollow cylindrical structures about 25 nm in diameter that serve as tracks for the long-range bidirectional movement of intracellular membranous organelles and macromolecular cargo complexes. In axons, this movement is known as axonal transport [3]. The cargoes of axonal transport are conveyed by microtubule motor proteins: kinesins in the anterograde direction (towards the axon tip), and dyneins in the retrograde direction (towards the cell body) [4]. Neurofilaments, which are the intermediate filaments of nerve cells, are flexible rope-like polymers that measure about 10 nm in diameter [5]. These polymers function as space-filling structures that expand axonal cross-sectional area, thereby maximizing the rate of propagation of the nerve impulse [6, 7]. In large axons, neurofilaments are the single most abundant structure and occupy most of the axonal volume [8]. Mutant animals that lack neurofilaments develop smaller caliber axons and exhibit delayed conduction velocities [9–11].

In addition to their structural role in axons, neurofilaments are also cargoes of axonal transport, moving along microtubule tracks powered by kinesin and dynein motors [12–16]. The filaments move at rates similar to membranous organelles but the movements are less frequent, resulting in a “stop and go” motile behavior characterized by short bouts of movement interrupted by prolonged pauses on a time scale of seconds or shorter [17, 18]. The net result is an average rate of transport that is much slower than that for many other cargoes.

Neurofilaments have been observed to accumulate abnormally in axons in many neurodegenerative diseases including amyotrophic lateral sclerosis, hereditary spastic paraplegia, giant axonal neuropathy and Charcot-Marie-Tooth disease (also known as hereditary distal motor and sensory neuropathy) [5, 19–23], and also in many toxic neuropathies [24–28]. In extreme cases, these accumulations can lead to giant balloon-like axonal swellings [29–34]. These accumulations are thought to be caused by alterations in neurofilament transport, but the mechanism is not understood [35–40].

**Fig 1:**
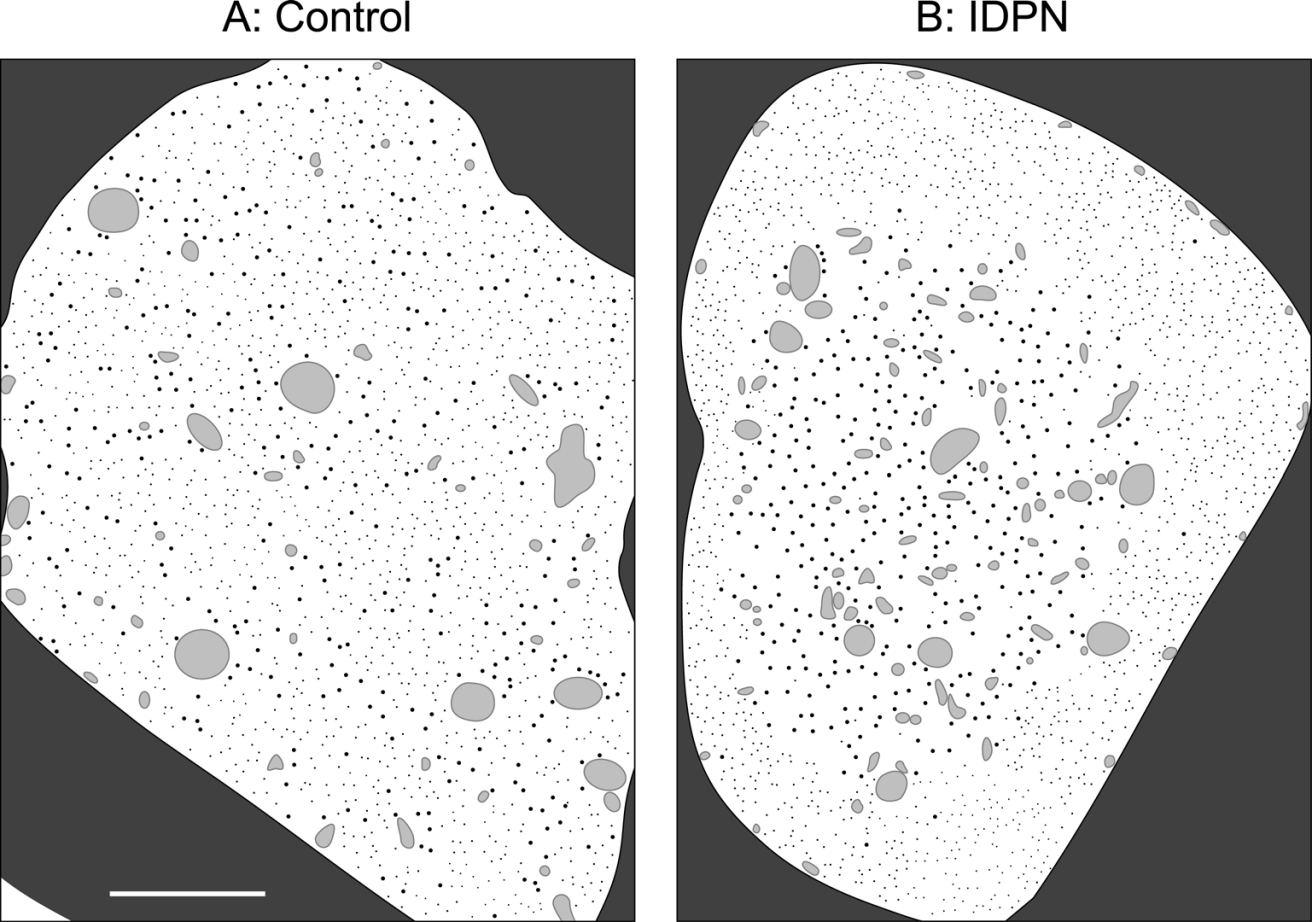
IDPN-induced segregation of microtubules and neurofilaments in axonal cross-sections. We show here drawings that are based loosely on the electron micrographs in Fig 3 in the reference Papasozomenos et al. [50]. Due to copyright restrictions we are not able to show the actual micrographs. The authors administered IDPN in physiological saline to adult male rats by intraperitoneal injection (2 mg/g body weight). Control injections consisted of physiological saline alone. For the micrographs on which these drawings are based, the animal was sacrificed after 2 weeks and the nerve was fixed and examined in cross-section by electron microscopy. Full experimental details can be found in the original article. (A) Drawing of a control axon in cross-section showing that the microtubules (large black dots), neurofilaments (small black dots) and membranous organelles (irregularly shaped grey blobs) are normally interspersed throughout the axonal cross-section. (B) Drawing of an axon in cross-section after IDPN treatment showing that the microtubules and organelles form a central core surrounded by a peripheral rim of neurofilaments. Note that the central core of microtubules and organelles contains very few neurofilaments and the outer rim of neurofilaments contains very few microtubules and organelles. The dark grey area outside of the axon is the myelin sheath. The scale bar is 1 *μ*m.

In healthy axons, microtubules and neurofilaments align along the length of an axon and are interspersed in axonal cross-sections [1, 41–43], with microtubules often forming small clusters in the vicinity of membranous organelles [8, 44, 45]. However, in many toxic and neurodegenerative disorders these polymers segregate, with microtubules and membranous organelles typically clustered in the center of the axon, and neurofilaments displaced to the periphery (Fig 1). This striking cytoskeletal reorganization, which is never observed in healthy axons, has been reported in neurodegenerative disorders as diverse as giant axonal neuropathy [46–48] and Charcot-Marie-Tooth disease [34, 49], as well as in neurotoxic neuropathies induced by exposure to agents as diverse as 2,5-hexanedione and 3,3’-iminodiproprionitrile (IDPN)[24, 50–57], aluminum [58], carbon disulfide [59, 60], estramustine phosphate [61], 1,2-diacetylbenzene [62] and 1,2,4-triethylbenzene [63], and in a transgenic mouse expressing a mutant neurofilament protein [64]. However, the mechanism of this segregation and its relationship to the neurofilament accumulation that also occurs in these different conditions is not known.

Microtubule-neurofilament segregation has been studied most extensively for IDPN and 2,5-hexanedione. IDPN is a compound closely related to the naturally occurring food poison 3-aminopropionitrile which causes the neurological disorder lathyrism [65–68], and 2,5-hexanedione is a metabolite of the industrial solvent hexane. The mechanism of toxicity is not known, but it is thought to involve chemical modification of neurofilaments, which presumably disrupts their normal interactions with microtubules in some way [25, 28, 69–74]. Systemic administration of IDPN or 2,5-hexanedione to rats by intraperitoneal injection or by addition to the drinking water causes selective impairment of neurofilament transport [75–79], focal accumulations of axonal neurofilaments leading to axon enlargement, and neurological defects similar to amyotrophic lateral sclerosis (ALS) in humans [80–83]. Sub-perineurial injection of IDPN or 2,5-hexanedione into peripheral nerves results in local microtubule-neurofilament segregation within just a few hours, preceding the accumulation of neurofilaments by hours or days [50–52, 56, 84]. This segregation does not appear to affect the axonal transport of membranous organelles, which continue to interact with and move along these tracks in spite of their clustering. Moreover, in the case of IDPN the segregation has been shown to be reversible [24, 50], as has the impairment of neurofilament transport [85]. In [24], a single injection of IDPN into rat sciatic nerves resulted in segregation in axons at the injection site within a few hours, but the segregation disappeared in about a day. In [50], a single injection of IDPN into the body cavity of rats resulted in segregation within the axons of the sciatic nerve after 4 days, and this disappeared after six weeks. Thus the microtubule-neurofilament segregation caused by IDPN and 2,5-hexanedione is fast, local and reversible.

Though the segregation of microtubules and neurofilaments in axons was first described more than 30 years ago, the underlying mechanisms are still poorly understood. Given that neurofilaments move along microtubule tracks and that microtubule-neurofilament segregation precedes neurofilament accumulation and axonal enlargement in rodent models, it is attractive to speculate that the segregation reflects an uncoupling of neurofilaments from their transport machinery [75]. However, the mechanism by which such an uncoupling at the molecular level might generate polymer segregation at the population level remains unclear.

To address these questions, we have developed a stochastic multiscale model for the cross-sectional organization of microtubules and neurofilaments in axons. The model describes microtubules, neurofilaments, and organelles as interacting particles that move in a 2D domain representing a cross-section of an axon, and incorporates axonal transports of neurofilaments and organelles, as well as volume exclusion and Brownian motion of all the particles. Neurofilaments and organelles dynamically bind to and unbind from nearby microtubules through molecular motors, and the motor cross-bridges are modeled as elastic springs. The longitudinal movement of neurofilaments and organelles along axons is modeled by stochastic addition and removal of these cargoes. The multiscale nature of the model lies in that it is built upon molecular processes that occur on a time scale of seconds or fractions of a second, and addresses segregation phenomena of two populations of polymers that occur on a time scale of hours to days.

Simulations of the model demonstrate that if we block neurofilament transport by preventing neurofilament from binding to microtubules, then organelles pull nearby microtubules together and gradually segregate them from neurofilaments on the same time course as observed in toxic neuropathies; while if we restore neurofilament transport, then microtubules and neurofilaments start to remix until their spatial distribution returns to normal. This suggests that the microtubule-neurofilament segregation observed in disease can be a consequence of the impairment of neurofilament transport. The model further predicts that (1) during the segregation process, microtubules first form small clusters, small clusters merge into bigger clusters, and eventually a single cluster forms close to the center of the domain; (2) in the absence of neurofilament transport, larger organelles are more effective in causing complete cytoskeletal segregation than small organelles with the same density. Further experimentation will be required to verify the insights and predictions of the model.

## Model

### The stochastic multiscale model

**Fig 2:**
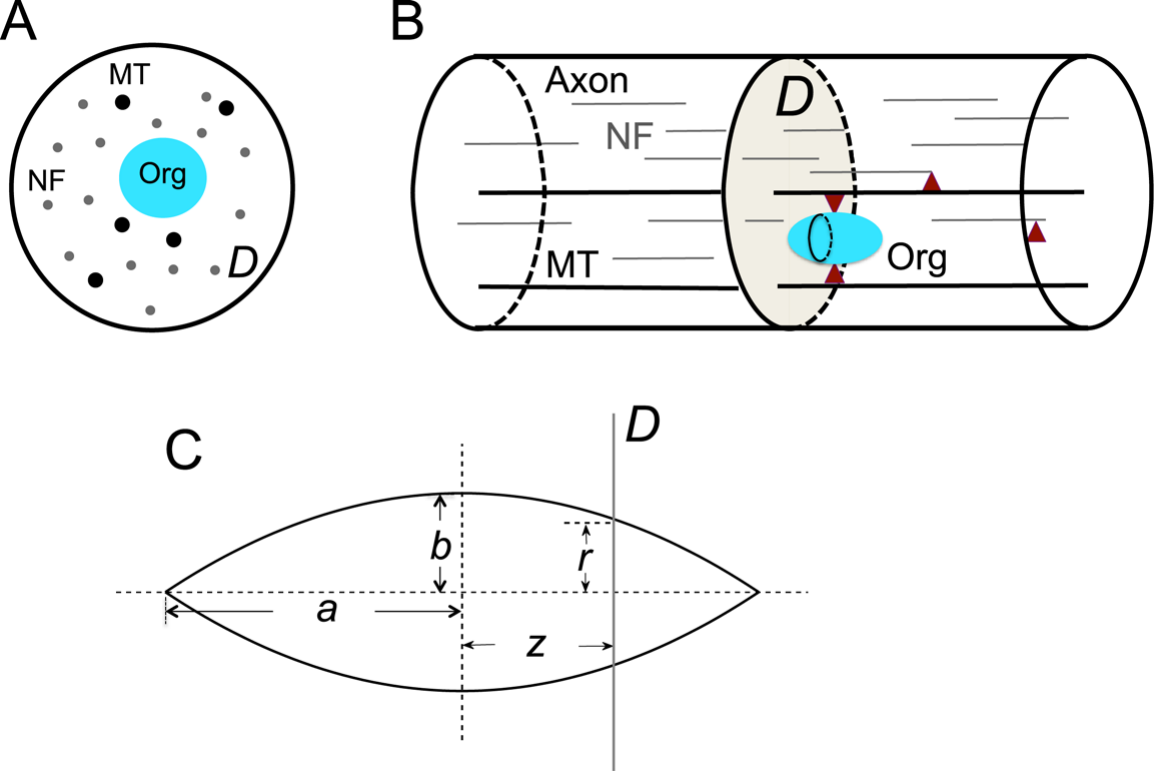
Model setup. (A) The model geometry. The computational domain *D* represents a cross-section of an axon. The black circle is the domain boundary representing the axon membrane. Small grey dots are neurofilaments (NF), large black dots are microtubules (MT) and cyan filled disks are organelles (Org). (B) The relation of *D* to the whole axon. Thin grey lines are neurofilaments, thick black lines are microtubules, the cyan body is an organelle, red triangles represent molecular motors that move microtubules and organelles along microtubule tracks. (C) The shape of organelles considered in this model. The cross-sectional radius of an organelle in *D* depends on its position relative to *D*.

In our model, microtubules, neurofilaments and organelles are described as individual particles that move in a circular domain *D* with fixed radius *R*_0_, representing a cross-section of an axon. Microtubules and neurofilaments are rod-like polymers that align along the length of axons, thus they are treated as nondeformable disks in *D* (Fig 2A), with center positions denoted by $x_{i}^{k}\;=\;(x_{i}^{k},\;y_{i}^{k})$ and radii by $r_{i}^{k}$. Here *k* = *M* or *N* is the index for particle type: *M* for microtubule, *N* for neurofilament; and *i* with 0 ≤ *i* ≤ *n* ^*k*^ is the index for the *k*-type particle where *n* ^*k*^ is the total number of *k*-type particles. The radii of microtubules and neurofilaments are constant, with $r_{i}^{M}=12.5\;\text{nm}$ and $r_{i}^{N}=5\;\text{nm}$. Organelles in axons have different sizes and shapes, and their cross-sectional geometry depends on their position relative to the cross-section (Fig 2B). In this model, we took organelles as spindle-shaped objects and, for simplicity, we did not consider possible shape changes (Fig 2C). Therefore the organelles exist as non-deformable disks in *D*, and as an organelle crosses *D*, its cross-sectional radius, $r_{i}^{O}$, varies according to its position, $z_{i}^{O}$, relative to *D*,

$$r_{i}^{O}=b(1-\frac{{\left(z_{i}^{O}\right)}^{2}}{a^{2}}),\;-a\leq z_{i}^{O}\leq a.$$ (1)

Here *a* is half of the organelle length, *b* is its maximum cross-sectional radius, $z_{i}^{O}$ is the distance of its center to *D*, and the index “O” stands for organelle. By varying the parameters *a* and *b*, we can vary the overall dimension of the organelles (Fig 2C).

We examined three key molecular mechanisms that contribute to the cross-sectional distribution of microtubules and neurofilaments: slow axonal transport of neurofilaments, fast axonal transport of organelles, and volume exclusion of all the particles. In the following sections we describe in detail how these mechanisms were incorporated into our model. We denote the unit vector pointing from $x_{i}^{k}$ to $x_{j}^{l}$ by ${\text{e}}_{ij}^{kl}$, and the surface distance between the *i*-th particle of *k*-type and *j*-th particle of *l*-type by $d_{ij}^{kl}$, given as,

$$d_{ij}^{kl}=|{\text{x}}_{i}^{k}\;-\;{\text{x}}_{j}^{l}|-r_{i}^{k}-r_{j}^{l}.$$ (2)

#### Mechanism 1: Slow axonal transport of neurofilaments

Neurofilaments interact with molecular motors (kinesin and dynein) which move these polymers along microtubules either anterogradely or retrogradely [14, 16, 86, 87]. The movements are fast but infrequent because the filaments spend most of their time pausing, which results in a slow average rate of transport [12, 88]. This longitudinal movement of neurofilaments along microtubules can change the spatial distribution of these polymers in axonal cross-sections. For example, if a neurofilament moves into the cross-section along a microtubule in a small cluster of microtubules it can displace one or more of the adjacent microtubules, dispersing the cluster. Alternatively, if a neurofilament between two microtubules moves out of the cross-section, then the two microtubules are able to diffuse closer together.

Based on the above considerations, we modeled neurofilament transport in the following way. A neurofilament in *D* can bind to a microtubule within a binding radius *R*_*b*_ with rates $k_{on}^{N}$. A neurofilament bound to a nearby microtubule can unbind with rate $k_{off}^{N}$, or move away and exit *D* with rate $k_{out}^{N}$. We conserved neurofilament number in *D* by replacing each departing neurofilament with a new entering neurofilament, placed at a distance *R*_*b*_ from a randomly selected microtubule. Neurofilaments and microtubules are long polymers that are aligned in parallel along the long axis of the axon so a new neurofilament can only enter *D* if a neurofilament was present at that location in the adjacent plane at the preceding time point. Thus, to prevent the entry of a new neurofilament in a region of *D* that is lacking other neurofilaments (specifically, this would be encountered when simulating the remixing of neurofilaments and microtubules after segregation), we only permitted the entry of a new neurofilament next to a microtubule that was already within a radius *R*_*b*_ of another neurofilament. We did not differentiate the direction of neurofilament movement along the axon because anterograde and retrograde movements have similar contributions to the distribution of neurofilaments in *D*. If a neurofilament is bound to a microtubule, they interact through the following elastic spring forces,

$${\text{G}}_{i,j}^{MN}\;=\;-{\text{G}}_{j,i}^{NM}\;=\;\kappa^{N}d_{ij}^{MN}{\text{e}}_{ij}^{MN}.$$ (3)

Here ${\text{G}}_{i,j}^{MN}$ and ${\text{G}}_{j,i}^{NM}$ are the forces acting on the *i*-th microtubule by the *j*-th neurofilament and vice versa, and *κ* ^*N*^ is the spring constant. If $d_{ij}^{MN}$ is bigger than the binding radius *R*_*b*_, then there is no spring force between the neurofilament-microtubule pair.

We assumed that each neurofilament could engage with only one microtubule at a time. The rationale for this is as follows. The on-rate and off-rate for neurofilament binding to microtubules is estimated to be 10^−2^/s and 6.5 × 10^−2^/s based on previous experiments. Thus a neurofilament within the binding radius of a microtubule would spend, on average, 1/(1 + 6.5) ≈ 0.13 of its time engaged with that microtubule, and the chance for one neurofilament within the binding radius of two microtubules to bind both simultaneously would be 0.13^2^ ≈ 0.017. Since in reality no neurofilament would remain within the binding radius of two microtubules at all times, the actual probability is even lower. Thus the chance for one neurofilament to interact with multiple microtubules simultaneously is negligible and we neglect it in our model.

#### Mechanism 2: Fast axonal transport of organelles

Like neurofilaments, membraneous organelles are also conveyed anterogradely or retrogradely along microtubule tracks by kinesin and dynein motors. However, these cargoes tend to spend much less time pausing, resulting in a much faster average rate of transport. Due to their large size, the movement of organelles can cause significant fluctuations of the microtubule and neurofilament organization by displacing these polymers laterally. These cargoes can bind multiple motors [89, 90] and, due to their large size, they can readily interact with multiple microtubules even if those microtubules are not close to each other [45, 91]. As an organelle moves along several microtubules, it can pull them closer together, similar to a “zipper”. This speculation is supported by *in vivo* data that demonstrate organelles being surrounded by multiple microtubules in close proximity [8, 44, 45, 91], and *in vitro* experiments [92] which show that motors bound to spherical cargoes can pull on multiple microtubules and align them.

Based on the above considerations, we modeled organelle movement in *D* in the following way. Organelles enter *D* randomly with rate $k_{in}^{O}$, moving along randomly chosen microtubules, and move persistently until they leave *D* completely. Thus each organelle is present in *D* for a time period that equals its length (2*a*) divided by its speed *s* ^*O*^, and

$$z_{i}^{O}=-a+s^{O}t,\;0\leq t\leq 2a/s^{O},$$ (4)

where *t* is the time the organelle has been present in *D*. As the organelle moves from one side of *D* to the other, its cross-sectional radius $r_{i}^{O}$ first increases from 0, reaches its maximum when it is halfway through, and then decreases to 0, which is given by Eq(1). While present in *D*, an organelle can bind stochastically to an available microtubule within a binding radius *R*_*b*_ with rate $k_{on}^{C}$ and unbind with rate $k_{off}^{C}$. If an organelle and a microtubule are bound, they interact through the linear spring force,

$${\text{G}}_{i,j}^{MF}\;=\;-{\text{G}}_{j,i}^{FM}\;=\;\kappa^{O}d_{ij}^{MF}{\text{e}}_{ij}^{MF}.$$ (5)

Here ${\text{G}}_{i,j}^{MF}$ and ${\text{G}}_{j,i}^{FM}$ are the forces acting on the *i*-th microtubule by the *j*-th organelle and vice versa, and *κ* ^*O*^ is the effective spring constant which represents the action of possibly multiple motors.

#### Mechanism 3: Volume exclusion

In addition to the active movement of neurofilaments and organelles and their interactions with microtubules through molecular motors, all the particles in the system interact through forces of volume exclusion.

Neurofilaments have sidearms which are highly-charged unstructured polypeptide domains. These sidearms project outward from the filament core to form an entropic brush that defines a zone of exclusion around the polymer via long-range repulsive forces [93–97], maximizing the space-filling properties of these cytoskeletal elements. Microtubule associated proteins such as tau also have highly charged long polypeptide domains that can have a similar volume-excluding effect [93, 98–100]. Based on these biological considerations, we modeled volume exclusion of neurofilaments, microtubules and organelles through the following pairwise repulsions,

$$R_{i,j}^{kl}=\{\begin{array}{ll} -\;\varepsilon^{kl}(L_{r}/d_{ij}^{kl}-1)\;e_{ij}^{kl} & \text{if}\;d_{ij}^{kl}\leq L_{r}\\ 0 & \text{if}\;d_{ij}^{kl}>L_{r} \end{array}.$$ (6)

Here ${\text{R}}_{i,j}^{kl}$ is the force acting on the *i*-th particle of *k*-type by the *j*-th particle of *l*-type, where *k*,*l* = *M*,*N* or *O*, 1 ≤ *i* ≤ *n* ^*k*^, and 1 ≤ *j* ≤ *n* ^*l*^. For example, ${\text{R}}_{i,j}^{MN}$ is the force acting on the *i*-th microtubule by the *j*-th neurofilament. Here *L*_*r*_ is the maximum interaction distance; *ε* ^*kl*^ specifies the magnitude of the force; and the negative sign preceding *ε* ^*kl*^ indicates that the force is repulsive. We note that this force goes to infinity as the surfaces of two particles approach each other and remains zero if the distance between two particles is larger than *L*_*r*_. The functional form of the force is similar to those used in [101, 102] for neurofilament repulsions and matches recent experimental data [103].

To keep all the particles inside the domain, we modeled volume exclusion of the particles with the domain boundary in a similar way. The force acting on the *i*-th particle of *k*-type by the axonal membrane is given by

$$R_{i}^{kB}=\{\begin{array}{ll} -\varepsilon^{kB}(L_{r}/d_{i}^{kB}-1)e_{i}^{kB} & \text{if }d_{i}^{kB}\leq L_{r}\\ 0 & \text{if }d_{i}^{kB}>L_{r} \end{array}.$$ (7)

Here the index *B* stands for “boundary”, $d_{i}^{kB}\;=\;R_{0}-|{\text{x}}_{i}^{k}|-r^{k}$, and ${\text{e}}_{i}^{kB}$ is the unit vector pointing from the center of the domain to ${\text{x}}_{i}^{k}$.

Microtubules, neurofilaments, and organelles can also interact with each other hydrodynamically through the axoplasm. Organelle movement can cause significant flow of the axoplasm near their surfaces and displace nearby microtubules and neurofilaments. As an organelle pushes into *D*, its radius increases and it pushes nearby fluid and particles away from itself; as it moves away from *D*, instead of leaving void behind it, it creates negative pressure which draws the axoplasm to flow back and fill the space. The hydrodynamic effect due to the movement of microtubules and neurofilaments is presumably smaller given their constant and smaller size in cross-section. In this model, we do not model the hydrodynamic interactions among these particles explicitly, but include this effect by adjusting the force prefactors associated with organelles. Specifically, when an organelle push into the domain, we double *ε* ^*kO*^ and *ε* ^*Ok*^ to take into account the contribution of the fluid flow it creates.

#### Model equations

The movements of microtubules, neurofilaments, and organelles in axons are viscous-dominated and thus inertia can be neglected. Under this simplification, we have the following system of stochastic differential equations

$$\begin{array}{l} \text{d}x_{i}^{k}\;=\;F_{i}^{k}/\mu^{k}dt+\sigma_{k}\text{d}W_{i}^{k},\;1\leq i\leq n^{k},\;\;k=M,\;N,\;F. \end{array}$$ (8)

Here $F_{i}^{k}$ is the sum of all applied forces on that particle specified in Eqs (3), (5), (6) and (7). For example, $F_{i}^{N}=\sum_{j}{\text{G}}_{i,j}^{NM}+\sum_{j}{\text{R}}_{i,j}^{NM}+\sum_{j,\;j\neq i}{\text{R}}_{i,j}^{NN}+\sum_{j}{\text{R}}_{i,j}^{NO}+{\text{R}}_{i}^{NB}.$ The constant *μ* ^*k*^ denotes the drag coefficient of the *k*-type particle. Finally ${\text{W}}_{i}^{k}$ are independent 2D Wiener processes modeling the random motion of these particles, and the amplitude *σ*_*k*_ is given by $\sigma_{k}=\sqrt{2D_{k}}$, where *D*_*k*_ is the diffusion coefficient of the particle calculated by the Einstein relation.

#### Simplifying assumptions of the model

We summarize the simplifying assumptions of the model below to help the readers to understand the application scope of the model.

1. The model tracks the cross-sectional movement of microtubules, neurofilaments, and organelles but does not distinguish their anterograde and retrograde movement. This is based on the consideration that the directionality of their movement along an axon has little effect on their cross-sectional distribution.
2. The model assumes that the total number of neurofilaments is conserved in the domain. This is based on the fact that their segregation from microtubules occurs on a time scale of hours, whereas their accumulation occurs on a much longer time scale of days.
3. We assumed that each neurofilament can only bind to one microtubule at a time and neglected the possibility of simultaneous interaction with multiple microtubules. The justification for this assumption is that neurofilaments spend only a small proportion of their time interacting with microtubules, so the chance of a single neurofilament interacting with two microtubules at the same time is likely to be very low. However, this assumption is not essential and has little effect on the results.
4. We assumed that organelles are non-deformable objects with a spindle shape, for the simplicity of computation.
5. We assumed that the movements of all the particles are dominated by viscous interactions and thus we neglected inertia. We did not explicitly incorporate the flow of the axoplasm around the moving particles and their hydrodynamic interactions.
6. We did not consider processes such as microtubule or neurofilament cross-linking through other proteins, e.g. [104–106], because there is no experimental data that support the presence of these mechanisms in the situation we consider here.

### Parameter estimation and simulation algorithm

The parameters used in our model are physical, and thus they are all measurable. Most of them have already been measured [107–115], or there exist experiments that can be used to estimate them. Table 1 summarizes all the parameter values, and the detailed methods to obtain these parameters are given in the S1 Text. The units of these parameters reflect the time scales for the molecular processes integrated into the model, which are seconds or fractions of a second.

**Table 1:** Model parameter values.

Parameter	Description	Values	Notes and Refs
*r* ^*N*^	Radius of neurofilament backbone	5 nm	[3, 107, 108]
*r* ^*M*^	Radius of microtubule backbone	12.5 nm	[3]
*r* ^*M*^	Radius of organelles	25–200 nm	[50, 109] E.E.
*R*_*b*_	capturing radius for microtubule-cargoe active binding	80 nm	[110]
$k_{on}^{N}$	rate for neurofilament binding	1.0 × 10^−2^ /s	[111], E.E.
$k_{off}^{N}$	rate for neurofilament unbinding	6.5 × 10^−2^ /s	[111]
$k_{out}^{N}$	rate for neurofilament departure	0.1 /s	E.E
$k_{on}^{O}$	rate for organelle binding	2 /s	[112, 113]
$k_{off}^{O}$	rate for organelle unbinding	2 /s	[112]
$k_{in}^{O}$	rate for organelle passage	0.105 /s	[50, 109], E.E.
*s* ^*O*^	speed of organelle movement along microtubules	1 *μ*m/s	[114]
*L*_*r*_	characteristic repulsion distance	121.2 nm	[102], E.E.
*ε*_*r*_	repulsion scale (= *ε* ^*NN*^)	0.5 pN	E.E.
*κ* ^*N*^	effective spring constant for microtubule-neurofilament binding	0.18 pN/nm	[115], E.E.
*κ* ^*O*^	effective spring constant for organelle-microtubule binding	0.9 pN/nm	[115], E.E.
*μ* ^*N*^	drag coefficient of neurofilaments	73.5 pN ⋅ s/*μ*m	E.E
*μ* ^*M*^	drag coefficient of microtubules	512 pN ⋅ s/*μ*m	E.E
*μ* ^*O*^	drag coefficient of organelles	40.3 pN ⋅ s/*μ*m	E.E
*D*_*N*_	diffusion coefficient of neurofilaments	5.59 × 10^−5^ *μ*m^2^/s	E.E.
*D*_*M*_	diffusion coefficient of microtubules	8.02 × 10^−6^ *μ*m^2^/s	E.E.
*D*_*F*_	diffusion coefficient of organelles	1.02 × 10^−4^ *μ*m^2^/s	E.E.

E.E.: estimated from experiments; see S1 Text for detailed information.

To solve the model numerically, we treated the binding and unbinding, arrival and departure of cargoes explicitly at discrete time steps, and integrated the model system Eq (8) using the explicit Euler’s method. Because *σ*_*k*_, *k* = *M*, *N*, *C* are constant in time, the numerical integrator has strong order 1.0 [116]. We chose a time step *h* much smaller than all the time scales involved in Mechanisms 1–3. For the simulations of segregation and remixing over hours to a day, we used *h* = 1/50 sec if there was no organelle in *D*, and *h* = 1/1600 sec otherwise in order to deal with the stiffness of the equations introduced by the pushing of organelles when they move into *D*. The detailed simulation algorithm is included in the S1 Text. The computational tool is written in C++.

## Results

### The organization of neurofilaments in normal axons

Morphometric studies suggest that neurofilaments are spaced randomly in axonal cross-sections when packed at low densities, but as the density increases they start to experience the volume-exclusionary repulsive forces of their neighbors and assume a less random distribution characterized by a more even neurofilament spacing [41, 43, 102]. In this section we demonstrate that the neurofilament distribution generated using our model agrees well with these experimental data.

Different methods have been used to characterize neurofilament distribution in axonal cross-sections. Kumar et al [102] used the radial distribution function (RDF) (also known as the pairwise correlation function). The RDF, denoted as *g*(*r*) describes how density varies as a function of distance from a reference particle. For particles that move randomly and completely independently, *g*(*r*) is a constant value of 1; while for crystalline structures *g*(*r*) forms peaks at precisely defined intervals. For neurofilaments in axons the shape of *g*(*r*) typically lies between these two extremes, increasing sharply from 0 and forming a peak around 30 − 50 nm [102]. Another method used often is to calculate the occupancy probability distribution (OPD), which is the distribution for the number of particles within an observation window of a specified shape and size [41, 43, 102]. For neurofilaments, the OPD can be approximated by Guassian [43, 102].

In previous experimental studies, the RDF and OPD of neurofilaments were calculated in selected regions of axonal cross-sections with almost no microtubules and organelles. To mimic such conditions, we performed simulations with exclusively neurofilaments, i.e., *n* ^*M*^ = *n* ^*O*^ = 0, and thus the only acting mechanisms are the pairwise repulsions and the Brownian motion of neurofilaments. We used a square domain with side length 1*μ*m, and to minimize the effect of the boundary we used periodic boundary conditions. Under such conditions, the system Eq(8) reduces to

$$\text{d}{\text{x}}_{i}^{N}=\underset{j,\;j\neq i}{\sum }{\text{R}}_{i,j}^{NN}/\mu^{N}\text{d}t+\sigma_{\text{N}}\text{d}{\text{W}}_{i}^{N},\;1\leq \text{i}\leq {\text{n}}^{\text{N}}.$$

We initially put neurofilaments on a hexagon lattice inside the domain, and then “randomized” the distribution by simulating the model for sufficient time to observe no further change in the OPD or RDF. To solve the model, we used the explicit Euler’s method with a time step *h* = 1/200 sec.

**Fig 3:**
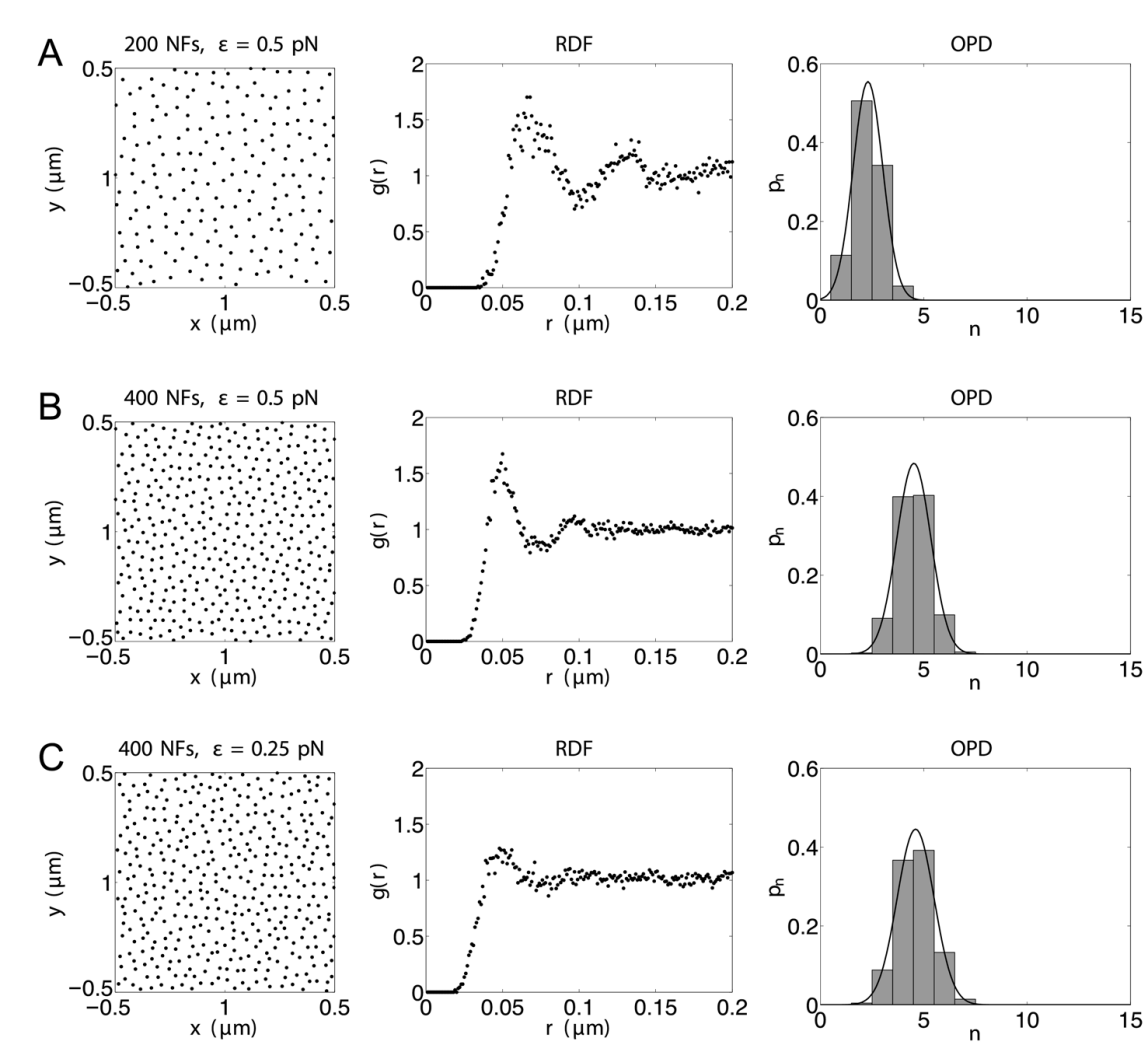
Simulated neurofilament distributions with different densities and repulsion magnitudes. Left: snapshots of neurofilament positions after randomizing for 25 sec. Middle: the radial distribution functions (RDF, *g*(*r*)). Right: the bars are histograms of the occupancy probability distribution (OPD, *p*_*n*_) using randomly chosen circular windows with a radius 60 nm, and the black curves are their Gaussian fits. Middle and right plots represent averages over 50 time frames between *t* = 25 sec and *t* = 30 sec. In all cases, *ε* is short for *ε* ^*NN*^. (A) *n* ^*F*^ = 200, *ε* ^*NN*^ = 0.5 pN; (B) *n* ^*F*^ = 400, *ε* ^*NN*^ = 0.5 pN; (C) *n* ^*F*^ = 400, *ε* ^*NN*^ = 0.25 pN. Other parameters are the same as specified in Table 1.

We first investigated how the neurofilament distribution depends on its density. We took *ε* ^*NN*^ = 0.5 pN and used increasing neurofilament densities of 200 and 400 per *μ*m^2^ (Fig 3, Rows A and B). For each case, the left panel is a plot of the coordinates of the neurofilaments after randomizing for 25 sec; the middle panel is a plot of the RDF which represent averages over 50 time frames between 25 sec and 30 sec; and the right panel is a plot of the averaged OPD and its Gaussian fit. The methods that we used to calculate the RDF and OPD are the same as in [102] and described in the supporting information (S1 Text). These plots show that as the neurofilament density becomes higher, the separation of the peaks of the RDF becomes smaller, and the average and variance of the OPD becomes larger as the neurofilament density becomes larger. General features of these plots are in tight agreement with experimental data presented in [41, 43, 102].

The magnitude of the repulsion between two neurofilaments depends on the charges of their sidearms. As the phosphorylation level of their sidearms becomes higher, their mutual repulsion becomes larger. We next investigated how the neurofilament distribution depends on the effect of sidearm phosphorylation by fixing the neurofilament density and varying *ε* ^*NN*^. We took the neurofilament density to be 400 per *μ*m^2^, and *ε* ^*NN*^ to be 0.25 pN and 0.5 pN. Fig 3B and 3C shows that as *ε* ^*NN*^ becomes larger, the locations of neurofilaments become more regular, the peaks of the RDF are better defined, and the variance of the OPD becomes smaller.

### Impairment of neurofilament transport leads to microtubule-neurofilament segregation

To investigate the mechanism of microtubule-neurofilament segregation in axons, we compared our simulations to experimental data obtained for IDPN in laboratory animals. We focused on IDPN because there is published data on both the rate and reversibility of the segregation. When IDPN is administered transiently by local injection into peripheral nerves, segregation appears within 2–6 hours and then disappears within 24 hours [24, 50, 57].

Since neurofilament accumulation and axonal swelling occur on a much slower time course than the segregation, they can be ignored for the purposes of our current analysis. Therefore, for simplicity, we took *D* to be a disk with fixed radius *R*_0_ = 1 *μ*m, and set the total number of neurofilaments *n* ^*N*^ to be constant. Specifically, if a neurofilament that was engaged with a microtubule left *D*, then it was replaced by a new neurofilament that entered *D* by association with a new randomly chosen microtubule. The total number of microtubules and neurofilaments in the domain were determined based on the experimentally determined densities of 18/*μ*m^2^ and 115/*μ*m^2^, respectively [50]. We thus calculated *n* ^*M*^ by the formula $n^{M}=\text{floor}(18\pi R_{0}^{2})=56$ and similarly we obtained *n* ^*N*^ = 356. Here the function floor(*u*) is the largest integer that is smaller than *u*. We considered organelles with *b* = 140 nm and *a*/*b* = 10 (Fig 2C) based on experimental data. All the parameter values are summarized in Table 1 and the estimation methods are given in S1 Text.

**Fig 4:**
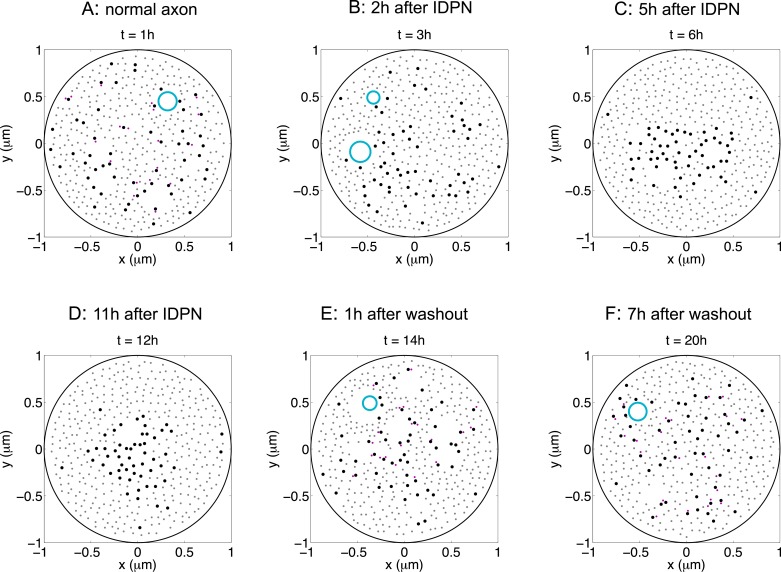
Reversible segregation of microtubules and neurofilaments in a single realization of the model. Neurofilament transport is blocked starting at *t* = 1 h and restored at *t* = 13 h. (A-F) Snapshots of the positions of microtubules, neurofilaments and organelles at *t* = 1 h, 3 h, 6 h, 12 h, 14 h, 20 h. All panels are from a single realization of the model. Large black dots are microtubules; small grey dots are free neurofilaments; small purple dots are neurofilaments engaged with microtubules; large cyan circles are organelles. (A) Microtubules and neurofilaments form a mixture under normal conditions. (B-D) Blockage of neurofilament transport leads to gradual segregation of microtubules and neurofilaments. (E,F) Restoration of neurofilament transport causes remixing of microtubules and neurofilaments. Parameters used: *n* ^*M*^ = 56, *n* ^*N*^ = 361. Neurofilament on-rate $k_{on}^{N}$ equals 0 between *t* = 1 h and 13 h. All other parameters are the same as in Table 1.

We started the simulations by including axonal transport of both neurofilaments and organelles, mimicking the conditions of normal axons. To distribute the neurofilaments and microtubules randomly without overlap, we first placed them on a hexagon lattice in *D* with no organelles, and then introduced volume exclusion and Brownian motion for enough time to randomize their positions. Starting from this initial condition, we then turned on the movement of both neurofilaments and organelles. Fig 4A is a snapshot of the simulated distribution of microtubules, neurofilaments, and organelles in a normal axon. The small grey dots are neurofilaments that are not engaged with microtubules, the small purple dots are neurofilaments that are engaged with microtubules, the large black dots are microtubules, and the large cyan circle is an organelle pushing into the cross-sectional domain. Note that a small fraction of the neurofilaments are bound to microtubules and moving along microtubules, that one microtubule can transport multiple cargoes (neurofilaments or organelles), and that one organelle can engage with multiple microtubules simultaneously.

We then blocked neurofilament transport selectively by resetting the binding rate of neurofilaments to microtubules, $k_{on}^{N}$, to be 0 at *t* = 1 h. This disengaged neurofilaments from their microtubule tracks and thus blocked their movement so that none could enter or leave *D*. Meanwhile, the transport of organelles was not affected: they continued to grab microtubules stochastically, pulling them together. This “zippering” effect caused the microtubules to gradually cluster (Fig 4B). By 6 hours, almost all the microtubules had migrated to the center of *D* and formed a single island surrounded by neurofilaments (Fig 4C and 4D). The central microtubule cluster contained organelles but relatively few neurofilaments whereas the peripheral zone of neurofilaments contained relatively few microtubules or organelles. This segregation pattern is strikingly similar to that observed in experiments and in disease, and the rate of segregation is comparable to that observed experimentally for local injection of IDPN into peripheral nerves of laboratory animals [24, 50].

After observing segregation, we restored neurofilament transport by resetting $k_{on}^{N}$ to its original value at *t* = 13 h. This immediately allowed neurofilaments on the periphery of the microtubule core within a distance *R*_*b*_ of a microtubule to bind to that microtubule stochastically and then either unbind or exit *D* after a short while, as dictated by their stop-and-go transport behavior. As explained in the Methods, each neurofilament that exited *D* was replaced with a new neurofilament seeded adjacent to a randomly selected microtubule, but only if that microtubule was within a distance of *R*_*b*_ from another neurofilament already in that plane. Over time this resulted in a gradual infiltration of neurofilaments into the microtubule cluster in a centripetal manner (i.e. from the outside edges progressing inward), leading to a gradual dispersal of the microtubules (Fig 4E) and a return to their normal interspersed organization (Fig 4F). These results agree tightly with previous experimental findings.

**Fig 5:**
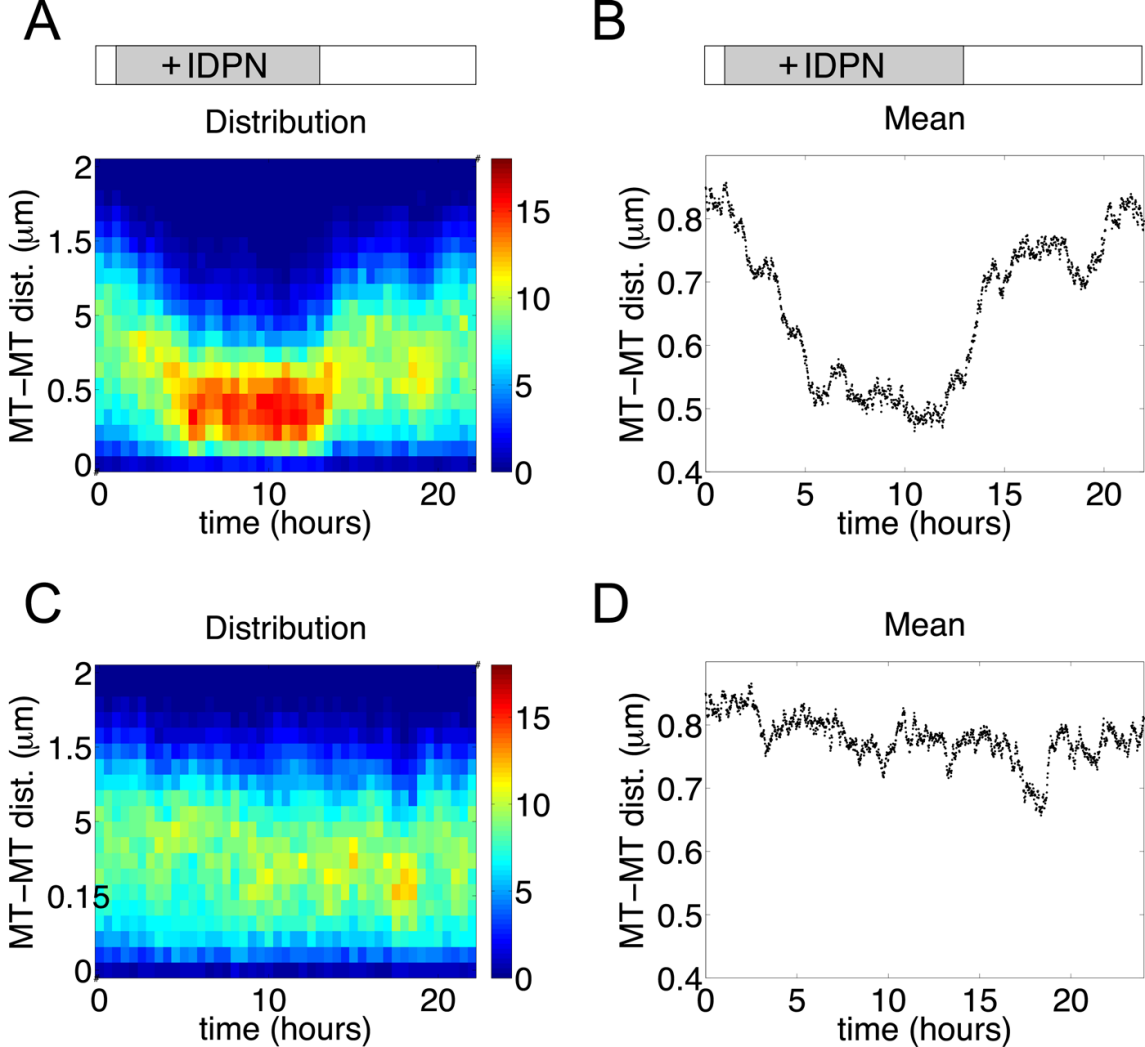
Statistics of the pairwise distances between microtubules (PDMT). (A, B) IDPN treatment started at t = 1 hour and stopped at t = 13 hours. (C, D) Control. (A, C) Distribution of the PDMT; data plotted for every 20 min. The pseudo color key represents the number of microtubule pairs. (B, D) Mean of the PDMT; data plotted for every min. Parameters used are the same as in Fig 4.

To characterize the reversible segregation of microtubules and neurofilaments, we plotted the distribution and the mean of the pairwise distance between two microtubules (PDMT) as a function of time. Fig 5A and 5B are plots calculated from the simulation shown in Fig 4, which demonstrate a significant progressive decrease of the PDMT upon elimination of neurofilament transport (*t* = 1h) and subsequent increase upon restoration of neurofilament transport (*t* = 13 h). Fig 5C and 5D are plots for a normal axon for comparison. We see that under normal conditions, because microtubules and neurofilaments are interspersed, the distribution of PDMT is broad and the mean of it is about 0.8*R*_0_; and as microtubules and neurofilaments segregate from each other, the distribution becomes more compact and the mean of the PDMT decreases by almost 40%.

Another way to incorporate blockage of neurofilament transport is to increase the off-rate of neurofilaments $k_{off}^{N}$. We performed simulations with $k_{off}^{N}$ 100 times larger, and obtained similar results as in Fig 4.

### Microtubule zippering by moving organelles is the causal mechanism for segregation

In the above section we have shown that in the absence of neurofilament transport, organelle transport leads to microtubule-neurofilament segregation. As we noted earlier, organelles can interact with multiple microtubules simultaneously and thus pull or zip nearby microtubules closer together. We next investigated the importance of this zippering mechanism for the segregation of microtubules and neurofilaments. To do this, we introduced a maximum number of microtubules that a single organelle can interact with simultaneously, denoted by *m*_max_, and investigated how the PDMT depends on *m*_max_ in the absence of neurofilament transport.

**Fig 6:**
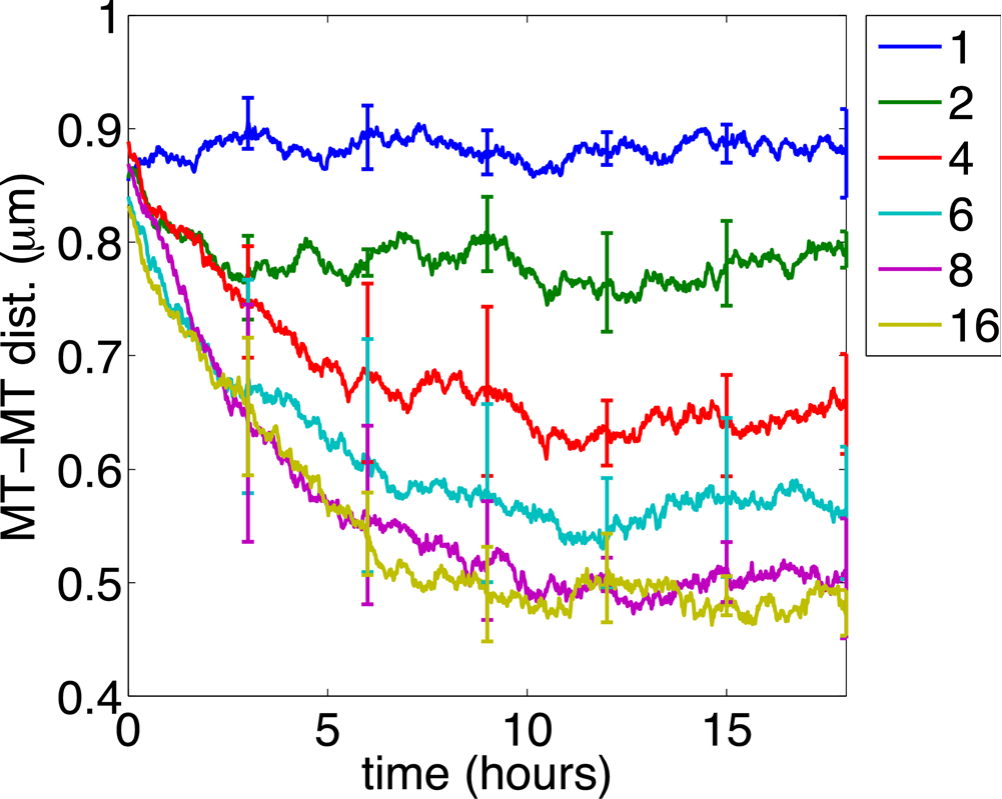
The effect of microtubule zippering by moving organelles. The mean of PDMT is plotted over time. The maximum number of microtubules that a single organelle can interact with simultaneously (*m*_*max*_) is set to be 1, 2, 4, 8, and 16 for the blue, green, red, cyan, purple, and yellow curves respectively. Each curve represents the average over 5 realizations of the model and the error bars are the standard deviation. All other parameters are the same as in Fig 4.

Fig 6 plots the mean of PDMT as a function of time given different values of *m*_max_. Each curve is averaged over five realizations with unpredictable seeds, and the error bars indicate the standard deviations over the realizations. If each organelle is only allowed to bind to one or two microtubules, i.e., *m*_max_ = 1 or 2, then microtubules and neurofilaments remain mixed over time and segregation does not occur at all (blue and green). Indeed, for *m*_max_ = 1, the mean of PDMT is slightly larger than that for a normal axon shown in Fig 5D. This is because organelles stir microtubules and neurofilaments and separate microtubules apart. As *m*_max_ increases, the PDMT curve decreases faster and the time needed to reach complete segregation decreases. Scatter plots of microtubules and neurofilaments (not shown here) show that for *m*_max_ = 4, partial but significant segregation was observed by 18 hours in all five realizations; for *m*_max_ = 6, complete segregation was observed by 18 hours in four out of five realizations; for *m*_max_ = 8 or 16, complete segregation was observed in all realizations within 10 hours. These results suggest that microtubule zippering by moving organelles is the causal mechanism for the segregation of microtubules and neurofilaments in the absence of neurofilament transport.

### Dependence on the size and the flux rate of organelles

**Fig 7:**
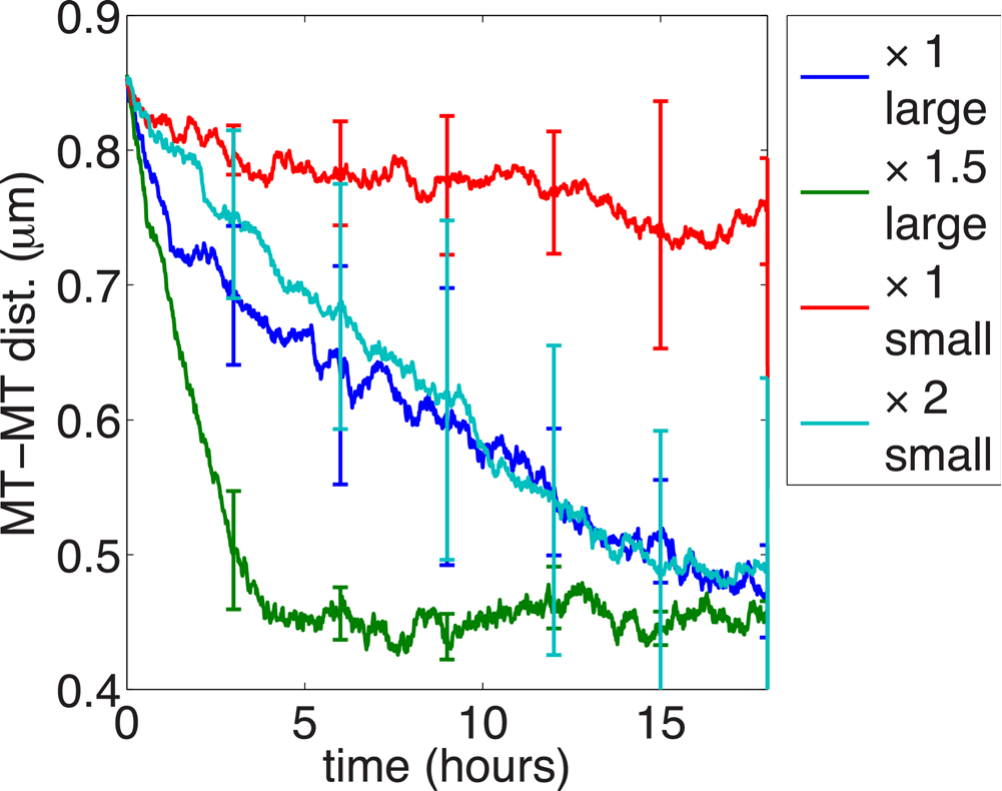
The segregation process depends on organelle size and flux rate. The mean PDMT is plotted over time. Organelle max radius *b*: 140 nm for blue and green curves (same as Fig 4); *b* = 70 nm for red and cyan curves. Organelle flux rate $k_{in}^{O}$: same as Fig 4 (x1; blue and red), 1.5-fold greater (x1.5; green) and 2-fold greater (x2; cyan). Each curve represents an average of 5 realizations of the model and the error bars are the standard deviation. All other parameters are the same as in Fig 4.

We next investigated the dependence of the segregation on the size and the flux rate of the organelles by simulating the model with different sizes of organelles, *b* = 140 nm or *b* = 70 nm, and different flux rates $k_{in}^{O}$. Fig 7 plots the mean PDMT over time for four situations: *b* = 140 nm and $k_{in}^{O}=0.105/\text{s}$ (shown in blue), which is the same as in Fig 4; *b* = 140 nm and $k_{in}^{O}=0.1575/\text{s}$ (shown in green); *b* = 70 nm and $k_{in}^{O}=0.105/\text{s}$ (shown in red); and *b* = 70 nm and $k_{in}^{O}=0.21/\text{s}$ (shown in cyan). These results suggest that (1) for organelles of the same size, the more frequently they move through *D*, the faster the segregation occurs; (2) given the same flux rate across *D*, larger organelles are more capable of clustering microtubules and segregating them from neurofilaments than small organelles, and this is because on average larger organelles can interact with more microtubules simultaneously.

**Fig 8:**
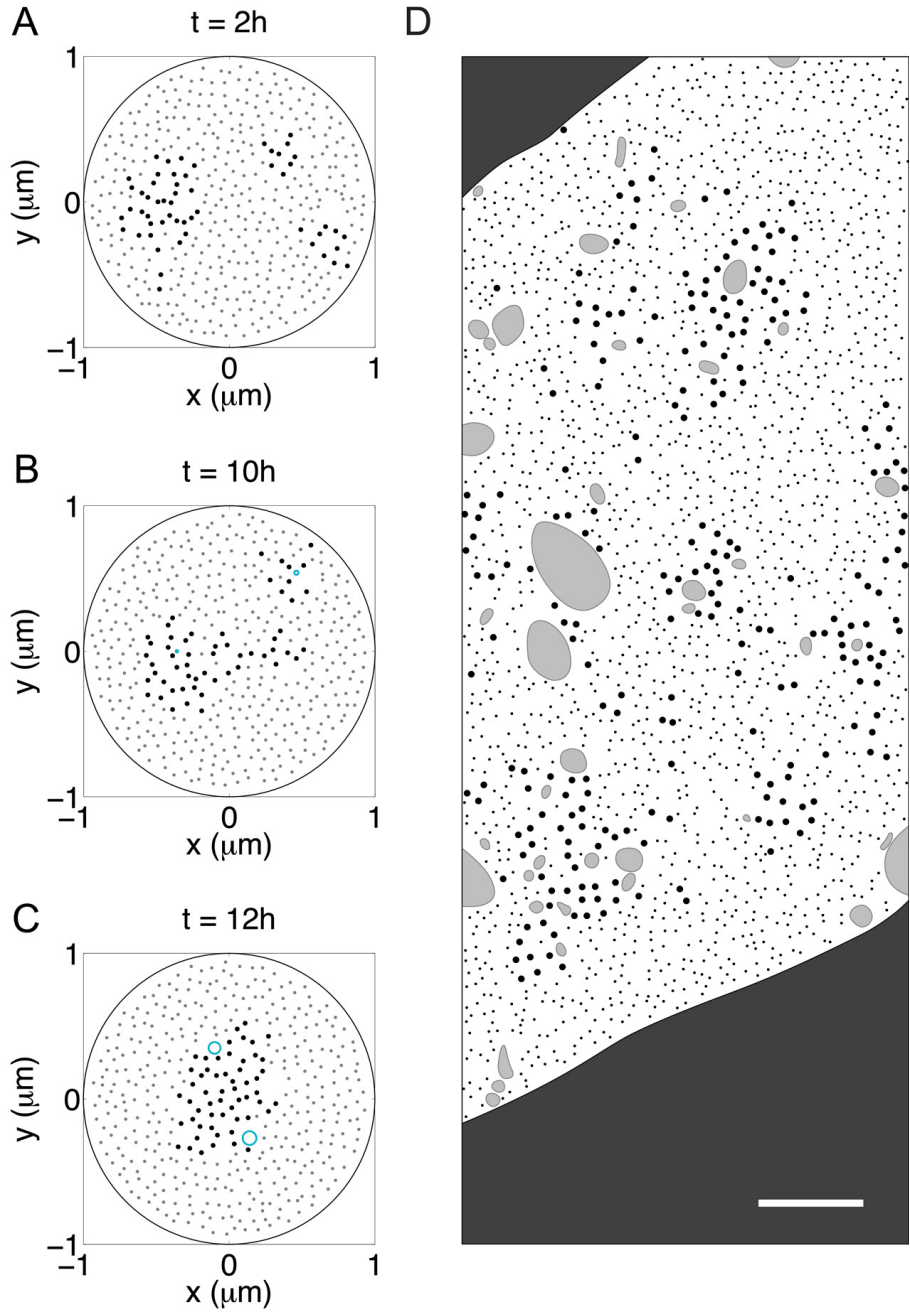
Segregation proceeds by the coalescence of microtubule islands. (A-C) Snapshots of the segregation process in a single realization of the model. Large black dots are microtubules, small grey dots are neurofilaments, and cyan circles are organelles. (A) Microtubules form three clusters by *t* = 4 h. (B) These clusters remain separated for several hours until two of them merge around *t* = 8 h. (C) Finally, all microtubules form a single big cluster near the center of the domain. The dimension of the organelles: *b* = 70 nm, *a*/*b* = 10. The flux rate of the organelles: 0.21 /s. All other parameters are the same as in Fig 4. (D) A drawing based loosely on the electron micrograph in Fig 9A of Zhu et al. [73], showing a cross-section of an L5 ventral nerve root axon from an animal that was exposed to IDPN. Due to copyright restrictions we are not able to show the actual micrographs. The authors administered IDPN in physiological saline to adult mice by intraperitoneal injection (1.5 mg/g body weight) and supplemented with 0.02% IDPN in the drinking water. For the micrograph on which this drawing is based, the animal was sacrificed 1 week after the first injection. Full experimental details can be found in the original article. Note the presence of multiple microtubule clusters, which resembles the intermediate stages of segregation in the simulations. Microtubules are represented by the large black dots, neurofilaments by the small black dots, and membranous organelles by the irregularly shaped grey blobs. The dark grey area outside of the axon is the myelin sheath. The scale bar is 0.4 *μ*m.

Interestingly, simulations of the model demonstrate that during the segregation process microtubules frequently form smaller clusters first, then these small clusters gradually merge with each other to finally form a single large cluster near the center of the domain. These intermediate states were more apparent in simulations with small organelles, presumably because the rate at which the smaller clusters merge is slower under this condition. Fig 8A–8C are snapshots of these intermediate states captured in a single realization (corresponds to the cyan curve in Fig 7). A similar pattern of isolated clusters of microtubules has also been reported by Zhu et al [73] (Fig 8D) (see Discussion).

### Partial blockage of neurofilament transport: dosage effect

**Fig 9:**
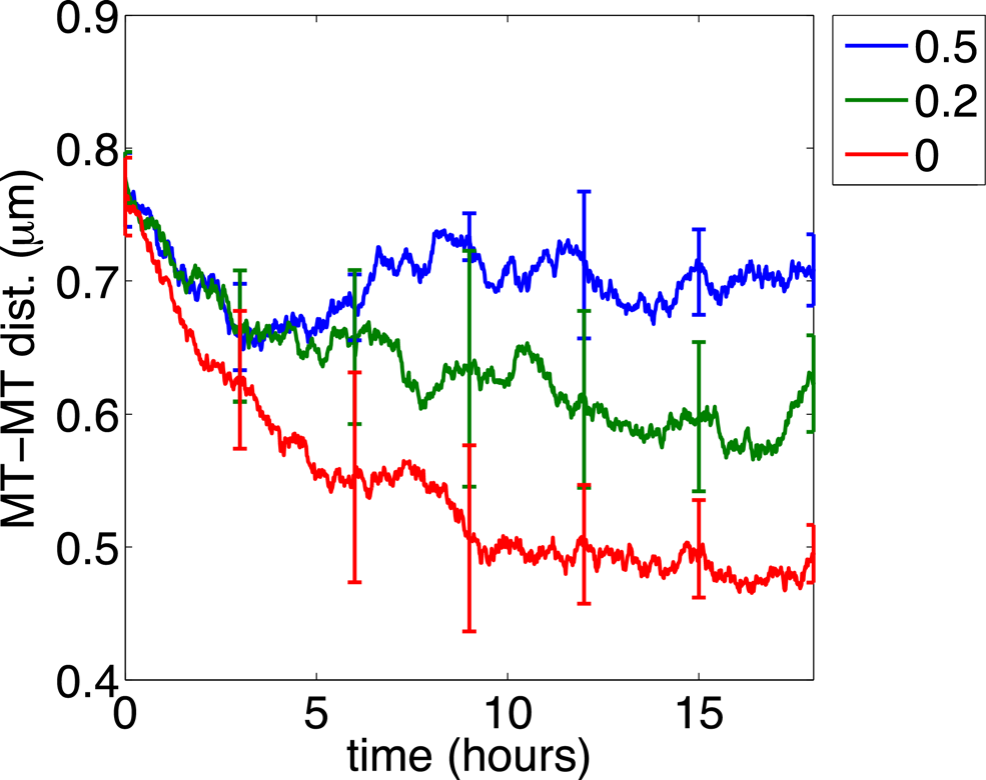
Dependence of microtubule-neurofilament segregation on $k_{on}^{N}$. Each curve plots the mean of the PDMT over time averaged over five realizations, and the error bars are the standard deviations. The rate $k_{on}^{N}$ is reduced to 50% (blue), 20% (green), and 0% (red) of the value in Fig 4 at *t* = 1 h. All other parameters are the same as in Fig 4.

We finally investigated the cross-sectional distribution of microtubules and neurofilaments when neurofilament transport is partially blocked. In the case of segregation induced by IDPN, this might be considered equivalent to lowering the IDPN concentration. To do this we reduced $k_{on}^{N}$ by different extents at *t* = 0h. Fig 9 plots the mean of PDMT over time for $k_{on}^{N}$ equals 0.5, 0.2 and 0 times of its original value. Each curve was obtained by averaging over 5 realizations with unpredictable seeds, and the error bars indicate the standard deviations about the mean. The data indicate that when $k_{on}^{N}$ is small enough, there is insufficient neurofilament transport to counteract the organelle-dependent microtubule clustering, and segregation is observed. However, as $k_{on}^{N}$ becomes larger, the rate of microtubule clustering becomes slower and the resulting clusters become less compact, reflecting less efficient segregation. Increasing $k_{off}^{N}$ has a similar effect: as $k_{off}^{N}$ becomes larger, the rate of microtubule clustering becomes faster and the clusters become more compact (not shown). Thus the rate and extent of microtubule-neurofilament segregation is dependent on the extent of inhibition of neurofilament transport.

## Discussion

### Summary of our model

We developed a novel stochastic multiscale model for the cross-sectional distribution of microtubules and neurofilaments in axons. The model describes microtubules, neurofilaments, and membranous organelles as interacting particles in an axonal cross-section. It incorporates detailed descriptions of key molecular processes that occur within seconds, including the axonal transport of neurofilaments and membranous organelles through this plane, as well as volume exclusion and Brownian motion of all the particles, and addresses the segregation phenomena that occur on a time scale of hours to days. The positions of the particles in the plane are governed by a system of stochastic differential equations.

Mathematical models of the axonal transport of neurofilaments and organelles have been developed previously to describe the longitudinal distribution of cargoes along axons [117–123]. However, those models were in 1D and did not consider the spatial arrangement and mechanical interactions of the cargoes and tracks in the radial dimension which are essential in understanding the segregation of microtubules and neurofilaments as well as the subsequent axonal swelling in neurological diseases. In our model, we describe in detail the dynamic interactions of neurofilaments, organelles, and nearby microtubules through molecular motors and volume exclusion in cross-section. Simulations of the model are in tight agreement with experimental data and generated a number of predictions that can be tested experimentally.

### Neurofilament and membranous organelle transports are competing processes

Simulations of the model demonstrate that if we block neurofilament transport selectively by preventing neurofilament binding to microtubules, while allowing organelle movement to continue, then the moving organelles tend to zipper nearby microtubules together so that they gradually segregate from the neurofilaments. The microtubule zippering action of the membranous organelles arises because we allow multiple motors to engage with a single organelle, which is consistent with experimental data and theoretical considerations [89, 90, 113, 124]. Restoration of neurofilament transport in the model allows the neurofilaments and microtubules to remix until their spatial distribution returns to normal. This suggests that neurofilament transport and organelle transport are competing processes in determining the cross-sectional distribution of microtubules: neurofilament transport can insert neurofilaments between adjacent microtubules, pushing those microtubules apart, while organelle transport can pull microtubules together when they move along multiple microtubules simultaneously, similar to a zipper. In normal axons, a dynamic balance between these two processes leads to the interspersed distribution of microtubules and neurofilaments, while in the absence of neurofilament transport, the microtubule zippering effect of organelle transport causes microtubules and neurofilaments to segregate. Thus our model predicts that the microtubule-neurofilament segregation that is observed in axons in neurotoxic and neurodegenerative diseases is a simple emergent property of the motile properties of membranous organelles that is triggered by selective impairment of neurofilament transport. An important and experimentally testable prediction of this study is that segregation is dependent on organelle movement. Further experimentation will be required to verify whether or not this prediction is correct.

### Why are the microtubule clusters mostly central?

An intriguing feature of microtubule-neurofilament segregation, which is consistent across all published reports, is that the microtubules generally cluster in the center of the axon, surrounded by a peripheral band of neurofilaments (see Introduction and Fig 1). It is interesting to note that this was usually the case in our simulations also. According to our model, the segregation generated by microtubule clustering is caused by an exclusion of neurofilaments from the microtubule domain due to their failure to interact. The central location of the microtubule bundle is essentially a boundary effect which arises because microtubules at the periphery of the axon can only be pulled towards microtubules that are located more centrally whereas microtubules in the center can be pulled towards microtubules on all sides. The net result is that microtubule zippering by moving organelles tends to pull these polymers towards the axon center, displacing the neurofilaments to the periphery. The organelles co-segregate with the microtubules because they must follow the available tracks.

### Segregation proceeds via the merging of small microtubule clusters

An interesting observation in our simulations is that microtubule-neurofilament segregation tends to proceed initially via the formation of small microtubule clusters that subsequently merge together. This was more apparent in simulations with smaller organelles, which are less efficient at zippering microtubules together (see discussion below). Multiple small microtubule clusters have been reported in some studies on microtubule-neurofilament segregation induced by IDPN [54, 73] (see Fig 8D), but there is no published time course of segregation so it remains to be proven that these clusters are indeed intermediate states. Interestingly, microtubule zippering in our simulations also gives rise to the formation of small microtubule clusters in healthy axons. However, with ongoing neurofilament transport these clusters are transient and rarely merge to form larger ones. This is consistent with reports that small clusters of microtubules, often adjacent to one or more membranous organelles, are commonly observed in electron micrographs of axons [8, 44, 45, 91].

### Factors influencing rate of segregation

Our analysis gives us some insights into the factors that influence the rate of microtubule-neurofilament segregation. First, given the same number density, larger organelles are more effective at causing segregation, because they can interact with more microtubules simultaneously and they can pull together microtubules that are farther apart. Second, segregation occurs faster if the flux rate of the organelles is larger. Third, segregation occurs faster if the degree of neurofilament transport impairment is larger. These predictions are experimentally testable. It is also clear that there must be some dependence on the density of motors on the organelle surface, as well as the neurofilament:microtubule ratio. We are currently performing an extensive investigation on how the segregation phenomena depend on combinations of the model parameters using model simplification, nondimensionalization and mathematical analysis. These efforts will provide further insight of the biological problem and will be published elsewhere in the future.

### The predicted rate of segregation is comparable to that in real axons

The best experimental data on the kinetics of microtubule-neurofilament segregation is for animals treated with the neurotoxin IDPN. However, the rate of segregation in animals treated with IDPN depends on the mode of administration. When applied systemically to rats using a single intraperitoneal injection, segregation was first noted after 4 days, and after 4 such injections at 3 day intervals, the resulting segregation persisted for 6–16 weeks [50]. In contrast, when applied locally at high concentration by sub-perineurial injection into peripheral nerve, microtubule-neurofilament segregation was evident after 2 hours, with the microtubule clusters becoming increasingly compact over the next 4–10 hours [24, 52]. Nagele et al. [57] analyzed the pairwise distance between microtubules (PDMT) and observed full compaction by 8 hours after injection. Sixteen hours later, segregation was no longer seen in most axons, indicating an almost complete reversal [52]. In our simulations, we observed segregation within 4–12 hours of a complete cessation of neurofilament transport, and remixing within 2–8 hours after a complete resumption of neurofilament transport. This rate of segregation is comparable to the kinetics observed experimentally for injections of IDPN into nerves, and suggests that this delivery method results in a transient but acute inhibition of neurofilament transport. We predict that the slower time course of segregation that is observed when IDPN is administered systemically is due to the lower effective dose experienced by the axons in those studies. The rate of remixing was a bit shorter in our simulations than in the experimental reports, which may be because we assumed an instantaneous recovery of neurofilament transport rather than a gradual one, which is more likely.

### What is the mechanism of neurofilament transport impairment?

It is important to note that the impairment of neurofilament transport that leads to microtubule-neurofilament segregation in toxic neuropathies and neurodegenerative diseases also leads eventually to focal neurofilament accumulations and axonal swellings (see Introduction). Since microtubules are the tracks along which neurofilaments move, and since microtubule-neurofilament segregation appears early and precedes neurofilament accumulation and axonal swelling, it has been hypothesized that the segregation reflects the uncoupling of neurofilaments from their transport machinery [24, 28]. Our modeling supports this hypothesis, but the molecular mechanism is unclear. Many of the neurotoxic agents that cause microtubule-neurofilament segregation and impair neurofilament transport (e.g. hexanedione, IDPN, carbon disulfide) are reactive molecules that could, or are known to, modify neurofilaments chemically [28]. It is thought that these compounds react with specific amino acid residues to form protein adducts which may then modify protein interactions, and that such chemical modifications target neurofilaments preferentially or that they somehow render these polymers more susceptible than other cargoes to transport impairments. This selectivity could arise, for example, due to the unique structure or unusual amino acid composition of neurofilament proteins. The mechanism of impairment could be by interfering with their interaction with molecular motors or with the interaction of these motors with the microtubule tracks. Future experimental studies will be required to resolve such questions.

### How do neurofilament accumulations arise?

The mechanism by which neurofilament accumulations arise is also of great interest given that this occurs in so many neurodegenerative diseases. Since local accumulations can only form if more neurofilaments move into a segment of axon than move out, the appearance of local swellings along axons implies some longitudinal instabilities in the transport of these cargoes. Therefore we propose that neurofilament segregation is an early event in neurofilament transport impairment but that longitudinal instabilities or non-uniformities in the transport impairment must arise to give rise to local accumulations and axonal swellings. We plan to address this in future studies. Due the complex spatial and temporal nature of this problem, which entails the interactions of multiple dynamic components, we believe that a full understanding can only be achieved by a combination of experimental and modeling approaches. Our present study is an important first step.

## Supporting Information

S1 Text A: calculation methods for the RDF and OPD in Fig 3. B: parameter estimation. C: simulation algorithm. (PDF)
