# Supplementary material for: A Stochastic Multiscale Model That Explains the Segregation of Axonal Microtubules and Neurofilaments in Neurological Diseases
Source: PLoS Comput Biol. 2015 Aug 18;11(8):e1004406. doi: 10.1371/journal.pcbi.1004406 (PMC4540448; doi:10.1371/journal.pcbi.1004406)
Supplement: S1 Text — (PDF) [file pcbi.1004406.s001.pdf]

# A Stochastic Multiscale Model that Explains the Segregation of Axonal Microtubules and Neurofilaments in Neurological Diseases Supporting Information

Chuan Xue* Blerta Shtylla† Anthony Brown‡

April 22, 2015

## 1 Supporting Information A: calculation methods for the RDF and OPD in Fig. 3

To calculate the RDF, for each time frame we used the centers of the neurofilaments in the domain $[-0.2\mu\text{m}, 0.2\mu\text{m}] \times [-0.2\mu\text{m}, 0.2\mu\text{m}]$ as reference points. We chose to use reference points in a smaller domain in order to avoid boundary effects. For each reference point we binned the center-to-center distances $(r)$ between the reference neurofilament and all other neurofilaments with a bin size $\Delta r = 1\text{nm}$, and normalized by the factor $2\pi r \Delta r \rho^N$ where $\rho^N$ is the density of neurofilaments in the whole domain. We then took the average over all reference points for each time frame and then over all time frames to obtain $g(r)$. We note that $g(r)$ is noisy due to under sampling and the small bin size (1 nm). Averaging over more time frames or using a larger bin size gave smoother $g(r)$. To compute the OPD $p_n$, we used circular windows with fixed radii 60 nm as in [1]. For each time frame, we sampled centers of $10n^N$ circles according to the uniform distribution in the domain $[-0.3\mu\text{m}, 0.3\mu\text{m}] \times [-0.3\mu\text{m}, 0.3\mu\text{m}]$ and calculated the particle occupancy number for each circle. We then produced a histogram of occupancy numbers obtained for all circles and normalized it by the total number of circles to obtain $p_n$.

## 2 Supporting Information B: parameter estimation

The parameters of the model are summarized in Table 1. In the following, we explain the methods used to estimate the parameters.

### Kinetic rates for neurofilament and organelle transports.

We assume that a neurofilament can only bind to a single microtubule at one time whereas an organelle, which is much larger, can interact simultaneously with one or more microtubules. The capturing radius $R_b$ for these interactions is taken to be 80 nm, which is the length of a kinesin motor when fully extended, as measured by electron microscopy [2].

*Department of Mathematics, Ohio State University, Columbus, OH 43220. Email: cxue@math.osu.edu
†Department of Mathematics, Pomona College, Claremont, CA 91711.
‡Department of Neuroscience, Ohio State University, Columbus, OH 43210.

The movement of neurofilaments along axons has been modeled as a bidirectional independent velocity jump process in 1D with two distinct pausing states in which a neurofilament either moves anterogradely or retrogradely, pauses for a short time, or pauses for a long time [3, 4]. Since that model is one dimensional, the kinetic rates extracted represent space averages over all neurofilaments in the axonal cross-section. For this reason, the kinetic rates for neurofilament transport in our model are related, but not identical, to the rates extracted in those other studies. We estimate $k^N_{on}$, the rate at which a neurofilament binds to a nearby microtubule within distance $R_b$ in our model, to be five times as large as the transition rate $\gamma_{01}$, the rate for a short-pause neurofilament to start moving, defined in [4]. We estimate $k^N_{off}$, the unbinding rate of neurofilaments in our model, to be the same as the rate $\gamma_{10}$, the rate for a moving neurofilament to stop and pause, as defined in [4]. Using $\gamma_{01} = 2.0 \times 10^{-3}$/s and $\gamma_{10} = 6.5 \times 10^{-2}$/s for myelinated axons, we obtain $k^N_{on} = 1.0 \times 10^{-2}$/s and $k^N_{off} = 6.5 \times 10^{-2}$/s.

The rate that a neurofilament leaves the domain, $k^N_{out}$, is estimated in the following way. The average time for a neurofilament to move through $D$ is $L_N/s^N$, where $L_N$ is the average length of moving neurofilaments and $s^N$ is the speed of the filament. Assuming that the neurofilament departure events are exponentially distributed then the rate $k^N_{out}$ is the reciprocal of the average time, i.e., $k^N_{out} = s^N/L_N$. Taking $L_N \approx 5\mu\mathrm{m}$, which is the approximate average length of moving neurofilaments in cultured neurons, and $s^N \approx 0.5\mu\mathrm{m/s}$ [5, 6], we obtain $k^N_{out} = 0.1\,\mathrm{s}^{-1}$.

The binding and unbinding rates of organelles to a nearby microtubule, $k^O_{on}$ and $k^O_{off}$, are assumed to be constant $2/\mathrm{s}$ which are comparable to the rates used in previous mathematical models of vesicular transport along microtubules [7, 8].

The passage rate for the organelles is calculated using their cross-sectional density. Assuming the cross-sectional organelle density is $\rho^O$, then the total number of organelles in $D$ is given by $\pi R_0^2 \rho^O$. Denoting the speed of the organelle along microtubule to be $s^O$ and the length of the organelle to be $2a$, then the time that an organelle remains in $D$ is given by $2a/s^O$. Assuming that organelle arrival is a Poisson process, then the Poisson rate can be calculated as $\pi R_0^2 \rho^O$ divided by $2a/s^O$, that is,

$$k^O_{in} = \pi R_0^2 \rho^O s^O/(2a).$$

The sizes and densities of axonal organelles have been most carefully studied in [9]. According to Table 1 in [9], there are two major sizes of organelles: large ones such as mitochondria have an average cross-sectional diameter of 280 nm, and small ones classified as tubular and vesicular profiles have an average cross-sectional diameter of 50 nm. The densities of these organelles were counted in longitudinal slices of axons, and need to be converted to cross-sectional densities for our model. We note that the cross-sectional density ($\rho^O$) and the longitudinal density (denote as $\rho^O_l$) are generally different. However, the area fraction occupied by the organelles averaged over cross sections and longitudinal sections are comparable, and this relation can be used to convert the longitudinal density to cross-sectional density. Assuming that the objects are cylinders with radius $r$ and length $l$, the area fractions measured in cross and longitudinal sections are given by $\pi r^2 \rho^O$ and $2rl\rho^O_l$ respectively. Equating these two, we obtain $\rho^O = 2l\rho^O_l/(\pi r)$. For the large organelles, $2r = 280\mathrm{nm}$, $l = 870\mathrm{nm}$, $\rho^O_l = 0.023/\mu\mathrm{m}^2$, and this leads to $\rho^O = 0.091/\mu\mathrm{m}^2$. For the small organelles, $2r = 50\mathrm{nm}$, $l = 180\mathrm{nm}$, $\rho^O_l = 0.02/\mu\mathrm{m}^2$, and this leads to $\rho^O = 0.0917/\mu\mathrm{m}^2$.

The density of organelles estimated above is comparable to the density data in published IDPN studies. For example, in Table 1 of [10], the mitochondrial density was measured to be $0.187 - 0.250/\mu\mathrm{m}^2$. Taking $\rho^O = 0.187/\mu\mathrm{m}^2$, $a = 10b = 2.8\mu\mathrm{m}$, $s^O = 1\mu\mathrm{m/s}$, and $R_0 = 1\mu\mathrm{m}$, we obtain $k^O_{in} = \pi R_0^2 \rho^O s^O/(2a) = 0.105/\mathrm{s}$.

## Parameters in the pairwise repulsion and elastic spring forces.

We first estimate $L_r$ which is the maximum distance for pairwise repulsions of particles. The parameter $L_r$ for neurofilament-neurofilament repulsion is approximately $2l^N$, where $l^N$ is the equilibrium brush thickness of the neurofilament sidearms. This was given by Eqn. (4) in [1], that is,

$$l^N = \left(\frac{12}{\pi^2}\right)^{1/3} Na \left(\frac{a}{s}\right)^{2/3}. \tag{S1}$$

Here $N$ is the number of amino acids per sidearm, $a$ is the length of an amino acid and $s$ is the spacing between neurofilament sidearms. Taking $N = 679$ [1], $a = 3.5$ angstroms [11, 12], and $s = 3\,\mathrm{nm}$ [1, 13], we obtain $l^N \approx 60.6\,\mathrm{nm}$ and thus $L_r = 121.2\,\mathrm{nm}$. For simplicity, we use the same $L_r$ for pairwise interactions of all kinds of particles and their interactions with the domain boundary.

We estimate the force prefactors $\varepsilon^{kl}$ in the following way. We assume that the repulsion force between two neurofilaments is approximately 1 pN when their surface distance is $d = 40\,\mathrm{nm}$. Under this assumption we have $\varepsilon^{NN} = 1/(L_r/d - 1)$. Taking $L_r = 121.2\,\mathrm{nm}$, we obtain $\varepsilon^{NN} \approx 0.5\,\mathrm{pN}$. We denote $\varepsilon^{NN}$ by $\varepsilon_r$ for simplicity of notation. We assume that the force prefactor for microtubule-microtubule, microtubule-neurofilament repulsions and repulsions of microtubules and neurofilaments with the boundary are the same as $\varepsilon_r$. For repulsions that involve organelles, we use a prefactor that is five times as large, that is, $\varepsilon^{kO} = \varepsilon^{Ok} = 5\varepsilon_r$.

Organelle movement can cause significant flow of the axoplasm near their surfaces and displace nearby microtubules and neurofilaments. As an organelle pushes into $D$, its radius increases and it pushes nearby fluid and particles away from itself; as it moves away from $D$, instead of leaving void behind it, it creates negative pressure which draws the axoplasm to flow back and fill the space. In this model, we do not include the hydrodynamic interactions among these particles explicitly, but include this effect by adjusting the force factors $\varepsilon$ associated with organelles. Specifically, when organelles push into the domain, we double $\varepsilon^{kO}$ and $\varepsilon^{Ok}$ to take into account the contribution of the fluid flow.

The effective spring constants $\kappa^N$ and $\kappa^O$ are calculated in the following way. The mean surface distance between a microtubule and a cargo engaged on it has been observed to be 17 nm (denote as $l_0$) [14, 15]. We assume that the spring force and the repulsive force between a microtubule-neurofilament pair equilibrates at $l_0$, i.e., $\kappa^N l_0 = \varepsilon_r(L_r/l_0 - 1)$. From this assumption we obtain

$$\kappa^N = \varepsilon_r(L_r/l_0 - 1)/l_0. \tag{S2}$$

Plugging the values of $\varepsilon_r$, $L_r$ and $l_0$ into this expression, we obtain $\kappa^N = 0.18\,\mathrm{pN/nm}$. Similarly, we assume that the spring force and the repulsive force of a microtubule-organelle pair equilibrates at $l_0$, and this leads to

$$\kappa^O = 5\varepsilon_r(L_r/l_0 - 1)/l_0, \tag{S3}$$

which is $\kappa^O = 0.9\,\mathrm{pN/nm}$. We note that the spring constant for a single kinesin motor is estimated to be $0.2 - 0.4\,\mathrm{pN/nm}$ in [16]. The spring constants here are different from the spring constant of a single motor in two ways: first, when a cargo moves along a microtubule there could be multiple motors being active, and thus the spring constants used here represent the sum of the spring constants over all active motors; second, the spring constants used here only take into account projections of the elasticity of individual motors in the plane orthogonal to the microtubule, whereas molecular motors are most likely slanted when dragging a cargo along a microtubule.

### Drag and diffusion coefficients

We treat neurofilaments as slender cylinders, and estimate the drag coefficient per unit length ($\mu/L$) by the formula given in [17], $\mu^N/L = 4\pi\eta/(\ln(L_N/b) + \ln 2 - 0.5)$, where $\eta$ is the viscosity of the axoplasm, $L_N$ is the characteristic length of the cylinder, and $b$ is the characteristic radius of the cross-section. The viscosity of the cell cytoplasm is estimated to be $\eta \sim 3 - 5\,\mathrm{pN}\cdot\mathrm{s}/\mu\mathrm{m}^2$ for mammalian cells [18, 19] . The persistence length of neurofilaments is 200-450 nm [20,21]. The characteristic radius of a neurofilament with its sidearms is about 20 nm [22]. Taking $L_N = 500\mathrm{nm}$ , $b = 20\mathrm{nm}$, we obtain $\mu^N/L = 4\pi\eta/(\ln(L_N/b) + \ln 2 - 0.5) \approx 14.7\,\mathrm{pN}\cdot\mathrm{s}/\mu\mathrm{m}^2$. Taking the length of the neurofilament to be $L = 5\mu\mathrm{m}$, we obtain $\mu^N \approx 73.5\,\mathrm{pN}\cdot\mathrm{s}/\mu\mathrm{m}$.

We estimate the drag coefficient for microtubule in a similar way. The persistence length of microtubules has been estimated to be $80 \pm 20\mu\mathrm{m}$ [23]. We estimate the characteristic radius of the microtubule with its associated proteins in axons to be 37.5 nm. Taking $\eta = 4\,\mathrm{pN}\cdot\mathrm{s}/\mu\mathrm{m}^2$, $L_M = 80\,\mu\mathrm{m}$ and $b = 37.5\,\mathrm{nm}$, we obtain $\mu^M/L = 6.4\,\mathrm{pN}\cdot\mathrm{s}/\mu\mathrm{m}^2$. We note that this estimate is close to the estimate obtained by treating microtubules as infinitely long cylinders, for which one can use the Oseen drag formula $\mu^M/L = 4\pi\eta/\log(4/R_e - \gamma + 0.5)$ where $\gamma \approx 0.5772$ is the Euler's constant and $R_e$ is the Reynolds number [24, 25]. The characteristic flow velocity in axonal cross-section is approximately $U = 0.2\mu\mathrm{m/s}$ given by the pushing of the organelles. Assuming that the density of the cytoplasm $\rho$ to be the same as water, we find that $R_e = 2Ub\rho/\eta \approx 4.3 \times 10^{-3}$. Plugging $\eta$ and $R_e$ into the Oseen's formula, we obtain the drag per unit length to be $6.3\,\mathrm{pN}\cdot\mathrm{s}/\mu\mathrm{m}^2$. Taking the sectional length of microtubule to be $L = 80\mu\mathrm{m}$, we obtain $\mu^M \approx 6.4 \times 80 = 512\,\mathrm{pN}\cdot\mathrm{s}/\mu\mathrm{m}$.

We estimate the drag coefficient for organelles using the formulas for a prolate ellipsoid given in [25]. Assume that the major axis of the ellipsoid is $2a$ and the minor axis is $2b$, then the drag coefficient per unit length is given by

$$\mu^O/L = \frac{16\pi\eta e^3}{2e + (3e^2 - 1)\ln[(1+e)/(1-e)]} \quad \text{for} \;\; 0 < e < 1,$$

where $e = \sqrt{(1 - (b/a)^2)}$ is the eccentricity. For $b/a = 0.1, 0.2$ and $0.5$, we have $\mu^O/L \approx 14.4, 17.9$ and $26.0\ \mathrm{pN}\cdot\mathrm{s}/\mu\mathrm{m}^2$. For organelles with $b = 140\mathrm{nm}$ and $b/a = 0.1$, we have $\mu^O \approx 40.3\mathrm{pN}\cdot\mathrm{sec}/\mu\mathrm{m}$.

We calculate the diffusion coefficients $D_k$ with $k = M, N$, and $O$ using the Einstein relation

$$D_k = k_B T/\mu_k, \tag{S4}$$

where $k_B$ is the Boltzmann's constant and $T$ is the absolute temperature. At room temperature (25 °C or 298 K), one has $k_B T = 4.11\mathrm{pN}\cdot\mathrm{nm}$. Using $\mu_N = 73.5\,\mathrm{pN\,s}/\mu\mathrm{m}$, we obtain $D_N \approx 5.6 \times 10^{-5}\,\mu\mathrm{m}^2/\mathrm{s}$. We calculated $D_M$ and $D_O$ in a similar way. Finally $\sigma_k$ is given by the relation $\sigma_k = \sqrt{2D_k}$.

## 3 Supporting Information C: simulation algorithm

To solve the model numerically, we chose a time step $h$ much smaller than all the time scales involved in Mechanisms 1-3, treated the binding and unbinding, arrival and departure of cargoes explicitly at discrete time steps, and integrated the model system (Eqn. 8 in main text) using the explicit Euler's method. Because $\sigma_k, k = M, N, C$ are constant in time, the numerical integrator has strong order 1.0 [26]. For the segregation simulations, we used $h = 1/50\,\mathrm{sec}$ if there was no organelle in $D$; otherwise we used $h = 1/1600\,\mathrm{sec}$ in order to deal with the stiffness of the equations introduced by the pushing of organelles when they move into $D$. The algorithm for a typical time step is summarized below.

**Algorithm for the model**

1. Stochastic removal of moving neurofilaments. For each moving neurofilament, generate a random number $r$ uniformly distributed in [0, 1]. If $r < 1 - e^{-k^N_{out}h}$ then remove it from $D$.

2. Update $z_i^O$ and $r_i^O$ for each organelle in $D$ according to Eqn. 1 and Eqn. 4 in the main text. If $z_i^O$ becomes bigger than or equal to $a$ then remove the $i$-th organelle and release all microtubules from it.

3. Stochastic unbinding of cargoes, i.e., neurofilaments and organelles, from their engaged microtubules. If a microtubule and an organelle are engaged, then generate a random number $r$ uniformly distributed in [0 1], and let them unbind if $r < 1 - e^{-k^O_{off}h}$. They also unbind if their surface distance is bigger than $R_b$. The same method is used to update microtubule-neurofilament binding.

4. Stochastic binding of cargoes to microtubules.

   (a) If the surface distance of a cargo and a microtubule is smaller than $R_b$, then generate a random number and determine if they intend to bind to each other. Loop through all microtubule-neurofilament and microtubule-organelle pairs, and find all potential binding events.

   (b) Accept or reject the potential binding events according to the availability of the associated microtubules in a random order. We assume that one microtubule has 5 tracks, each neurofilament occupies one track, an organelle with maximum radius $140\text{nm}$ occupies 2 tracks, and an organelle with maximum radius $70\text{nm}$ occupies 1.5 tracks.

5. Addition of new neurofilaments and organelles to $D$. The number of new neurofilaments equals the number that has been removed in step 1. The number of new organelles is determined using the rate $k^O_{in}$. Since the time step $h$ is much smaller than the mean arrival time of organelles $k^O_{in}$, we introduce a new organelle in each time step with probability $(1-e^{-k^O_{in}h})$. We then add these cargoes at $l_0 = 17\,\text{nm}$ away from a randomly chosen microtubule at a random angle. If the random location overlaps with an existing particle or the associated microtubule is too crowded, then a different microtubule and an angle is generated randomly. Once a new cargo is added, it is bound to the selected microtubule.

6. Update the positions of the microtubules, organelles and neurofilaments in $D$ by integrating the model system (Eqn. 8 in the main text) using the explicit Euler's method, i.e.,

$$\begin{aligned} \mathbf{x}_i^k(t+h) &= \mathbf{x}_i^k(t) + \mathbf{F}_i^k(t)h/\mu^k + \sigma_k \mathbf{r}_i^k \sqrt{h}, \\ 1 \le i &\le n^k, \quad k = M, N, F. \end{aligned} \tag{S5}$$

   where $\mathbf{r}_i^k$ are pairs of random numbers generated from the standard normal distribution using the BoxMuller transform [27]. To avoid large values of $\mathbf{r}_i^k$, they are regenerated if the absolute value of any component is greater than 5.

7. Go to Step 1 for the next time step.

# References


[1] S. Kumar, X. Yin, B. D. Trapp, M. E. Paulaitis, and J. H. Hoh. Role of long-range repulsive forces in organizing axonal neurofilament distributions: evidence from mice deficient in myelin-associated glycoprotein. *J Neurosci Res*, 68(6):681–690, Jun 2002.

[2] N. Hirokawa, K. K. Pfister, H. Yorifuji, M. C. Wagner, S. T. Brady, and G. S. Bloom. Submolecular domains of bovine brain kinesin identified by electron microscopy and monoclonal antibody decoration. *Cell*, 56(5):867–878, Mar 1989.

[3] Yinyun Li, Anthony Brown, and Peter Jung. Deciphering the axonal transport kinetics of neurofilaments using the fluorescence photoactivation pulse-escape method. *Phys Biol*, 11(2):026001, Apr 2014.

[4] Paula C. Monsma, Yinyun Li, J Daniel Fenn, Peter Jung, and Anthony Brown. Local regulation of neurofilament transport by myelinating cells. *J Neurosci*, 34(8):2979–2988, Feb 2014.

[5] L. Wang, C. L. Ho, D. Sun, R. K. Liem, and A. Brown. Rapid movement of axonal neurofilaments interrupted by prolonged pauses. *Nat Cell Biol*, 2(3):137–141, Mar 2000.

[6] L. Wang and A. Brown. Rapid intermittent movement of axonal neurofilaments observed by fluorescence photobleaching. *Mol Biol Cell*, 12(10):3257–3267, Oct 2001.

[7] Ambarish Kunwar, Michael Vershinin, Jing Xu, and Steven P. Gross. Stepping, strain gating, and an unexpected force-velocity curve for multiple-motor-based transport. *Curr Biol*, 18(16):1173–1183, Aug 2008.

[8] Robert P. Erickson, Zhiyuan Jia, Steven P. Gross, and Clare C. Yu. How molecular motors are arranged on a cargo is important for vesicular transport. *PLoS Comput Biol*, 7(5):e1002032, May 2011.

[9] R. S. Smith. The short term accumulation of axonally transported organelles in the region of localized lesions of single myelinated axons. *J Neurocytol*, 9(1):39–65, Feb 1980.

[10] S. C. Papasozomenos, L. Autilio-Gambetti, and P. Gambetti. Reorganization of axoplasmic organelles following beta, beta'-iminodipropionitrile administration. *J Cell Biol*, 91(3 Pt 1):866–871, Dec 1981.

[11] G. M. Malacinski. *Essentials of molecular biology*. Jones & Bartlett Learning, 2005.

[12] I. Mingarro, I. Nilsson, P. Whitley, and G. Von Heijne. Different conformations of nascent polypeptides during translocation across the ER membrane. *BMC cell biology*, 1(1):3, 2000.

[13] N. Geisler and K. Weber. Self-assembly in vitro of the 68,000 molecular weight component of the mammalian neurofilament triplet proteins into intermediate-sized filaments. *J Mol Biol*, 151(3):565–571, Sep 1981.

[14] R. H. Miller and R. J. Lasek. Cross-bridges mediate anterograde and retrograde vesicle transport along microtubules in squid axoplasm. *J Cell Biol*, 101(6):2181–2193, Dec 1985.

[15] J. Kerssemakers, J. Howard, H. Hess, and S. Diez. The distance that kinesin-1 holds its cargo from the microtubule surface measured by fluorescence interference contrast microscopy. *Proc Natl Acad Sci U S A*, 103(43):15812–15817, Oct 2006.

[16] C. M. Coppin, J. T. Finer, J. A. Spudich, and R. D. Vale. Measurement of the isometric force exerted by a single kinesin molecule. *Biophys J*, 68(4 Suppl):242S–244S, Apr 1995.

[17] RG Cox. The motion of long slender bodies in a viscous fluid. part 1. general theory. *J. Fluid Mech*, 44(part 3):790–810, 1970.

[18] I. M. Parhad, J. W. Griffin, D. L. Price, A. W. Clark, L. C. Cork, N. R. Miller, and P. N. Hoffman. Intoxication with beta, beta' -iminodipropionitrile: a model of optic disc swelling. *Lab Invest*, 46(2):186–195, Feb 1982.

[19] R. Swaminathan, C.P. Hoang, and A.S. Verkman. Photobleaching recovery and anisotropy decay of green fluorescent protein gfp-s65t in solution and cells: cytoplasmic viscosity probed by green fluorescent protein translational and rotational diffusion. *Biophysical Journal*, pages 1900–1907, January 1997.

[20] R. Beck, J. Deek, M. C. Choi, T. Ikawa, O. Watanabe, E. Frey, P. Pincus, and C. R. Safinya. Unconventional salt trend from soft to stiff in single neurofilament biopolymers. *Langmuir*, 26(24):18595–18599, Dec 2010.

[21] Oliver I. Wagner, Sebastian Rammensee, Neha Korde, Qi Wen, Jean-Francois Leterrier, and Paul A. Janmey. Softness, strength and self-repair in intermediate filament networks. *Exp Cell Res*, 313(10):2228–2235, Jun 2007.

[22] S.T. Hsieh, T.O. Crawford, and J.W. Griffin. Neurofilament distribution and organization in the myelinated axons of the peripheral nervous system. *Brain Research*, 642(1-2):316–26, 1994.

[23] M G L. Van den Heuvel, M. P. de Graaff, and C. Dekker. Microtubule curvatures under perpendicular electric forces reveal a low persistence length. *Proc Natl Acad Sci U S A*, 105(23):7941–7946, Jun 2008.

[24] Carl Wilhelm Oseen. Über die stokessche formel und über eine verwandte aufgabe in der hydrodynamik. *Ark. Mat. Astron. Fys*, 6(29):1–20, 1910.

[25] Allen T Chwang and Theodore Y Wu. Hydromechanics of low-Reynolds-number flow. Part 4. Translation of spheroids. *J. Fluid Mech*, 75(4):677–689, 1976.

[26] P. E. Kloeden and E. Platen. *Numerical solution of stochastic differential equations*, volume 23. Springer, 1992.

[27] George EP Box, Mervin E Muller, et al. A note on the generation of random normal deviates. *The Annals of Mathematical Statistics*, 29(2):610–611, 1958.
